# In-depth analysis of SARS-CoV-2–specific T cells reveals diverse differentiation hierarchies in vaccinated individuals

**DOI:** 10.1172/jci.insight.156559

**Published:** 2022-04-08

**Authors:** Li Li, Muharrem Muftuoglu, Shaoheng Liang, Mahesh Basyal, Jiangxing LV, Mehmet Emin Akdogan, Ken Chen, Michael Andreeff, Christopher R. Flowers, Simrit Parmar

**Affiliations:** 1 Department of Lymphoma and Myeloma,; 2 Department of Leukemia, and; 3 Department of Bioinformatics and Computational Biology, The University of Texas MD Anderson Cancer Center, Houston, Texas, USA.; 4 The University of Texas at San Antonio, San Antonio, Texas, USA.

**Keywords:** COVID-19, Immunology, Adaptive immunity

## Abstract

SARS-CoV-2 vaccines pose as the most effective approach for mitigating the COVID-19 pandemic. High-degree efficacy of SARS-CoV-2 vaccines in clinical trials indicates that vaccination invariably induces an adaptive immune response. However, the emergence of breakthrough infections in vaccinated individuals suggests that the breadth and magnitude of vaccine-induced adaptive immune response may vary. We assessed vaccine-induced SARS-CoV-2 T cell response in 21 vaccinated individuals and found that SARS-CoV-2–specific T cells, which were mainly CD4^+^ T cells, were invariably detected in all individuals but the response was varied. We then investigated differentiation states and cytokine profiles to identify immune features associated with superior recall function and longevity. We identified SARS-CoV-2–specific CD4^+^ T cells were polyfunctional and produced high levels of IL-2, which could be associated with superior longevity. Based on the breadth and magnitude of vaccine-induced SARS-CoV-2 response, we identified 2 distinct response groups: individuals with high abundance versus low abundance of SARS-CoV-2–specific T cells. The fractions of TNF-α– and IL-2–producing SARS-CoV-2 T cells were the main determinants distinguishing high versus low responders. Last, we identified that the majority of vaccine-induced SARS-CoV-2 T cells were reactive against non-mutated regions of mutant S-protein, suggesting that vaccine-induced SARS-CoV-2 T cells could provide continued protection against emerging variants of concern.

## Graphical abstract

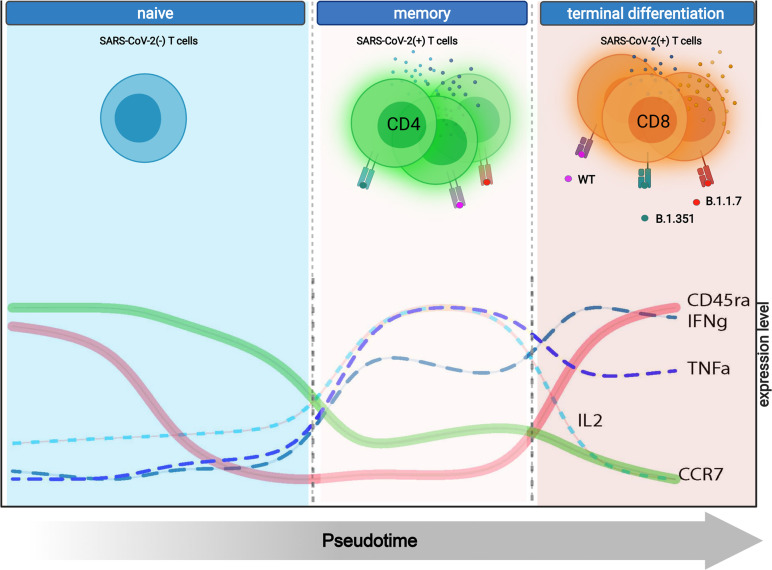

## Introduction

COVID-19 infection is a critical public health issue, and mRNA-based vaccines, mRNA-1273 (Moderna) and BNT162b2 (BioNtech/Pfizer), harboring modified RNAs spanning the whole length of spike (S-) protein, are highly effective in inducing protective cellular and humoral immunity conferring protection against COVID-19. High efficacy of mRNA-1273 and BNT162b2 vaccines in clinical trials suggests that mRNA-based vaccines induce a protective immune response in every individual, and correlative studies in these trials mostly relied on assessment of presence and titers of SARS-CoV-2–specific neutralizing antibodies (1, 2). The longevity of vaccination-induced adaptive and humoral immune response is a matter of intense debate. Previous experience with SARS-CoV-1 infection revealed that antibody responses waned over time and SARS-CoV-1–specific neutralizing antibodies were not detectable beyond 2 to 3 years (3). On the contrary, the adaptive immune response SARS-CoV-1 (etiologic agent of SARS) T cells mediated was maintained, and memory T cell responses specific for SARS-CoV-1 persisted more than a decade (4–6). Determining the breadth and durability of adaptive immunity, particularly cellular immune response against SARS-CoV-2, in vaccinated individuals could be of great importance since it could provide valuable insights into durability of vaccine-induced cellular immune response, infection risk, and the need for booster shots. Thus, it will be critical from a public health perspective to determine whether vaccinated individuals develop long-term memory T cell immunity against SARS-CoV-2.

The global emergence of various SARS-CoV-2 variants has raised concerns of increased transmissibility, virulence, and potential evasion of immune response elicited by vaccination or previous infection. Whether vaccine-induced adaptive immune response could confer continued protection against new variants is of crucial importance in adopting new strategies to control the COVID-19 pandemic and develop innovative strategies to tackle emerging SARS-CoV-2 variants. One particular concern is that accumulated mutations in the spike (S-) gene could lead to the emergence of new variants capable of evading adaptive immune response. Multiple variants of concern (VOCs) emerged globally over time and were detected in the United Kingdom (Alpha, B.1.1.7), South Africa (Beta, B.1.351), Brazil (Gamma, P.1), and India (Delta, B.1.617.2). Indeed, antibodies from vaccinated individuals were less effective against Beta, Gamma, and Delta variants (7, 8). This indicates that emerging VOCs could bypass the humoral arm of adaptive immunity. Therefore, whether any reduction in cellular adaptive immune response against emerging VOCs parallels the decline in antibody-mediated crossneutralization of novel VOCs in vaccinated individuals is a major concern that should be addressed in future studies.

Herein, we aimed to interrogate quantitative and qualitative features of SARS-CoV-2–specific T cell immunity induced by mainly mRNA-based vaccines, which could provide valuable insights into longevity of SARS-CoV-2–specific T cell–mediated immunity. We also utilized peptide pools spanning the mutated regions of variant S-protein to assess T cell response when recognizing mutant regions versus non-mutated regions.

## Results

### Study design.

**Figure 1:**
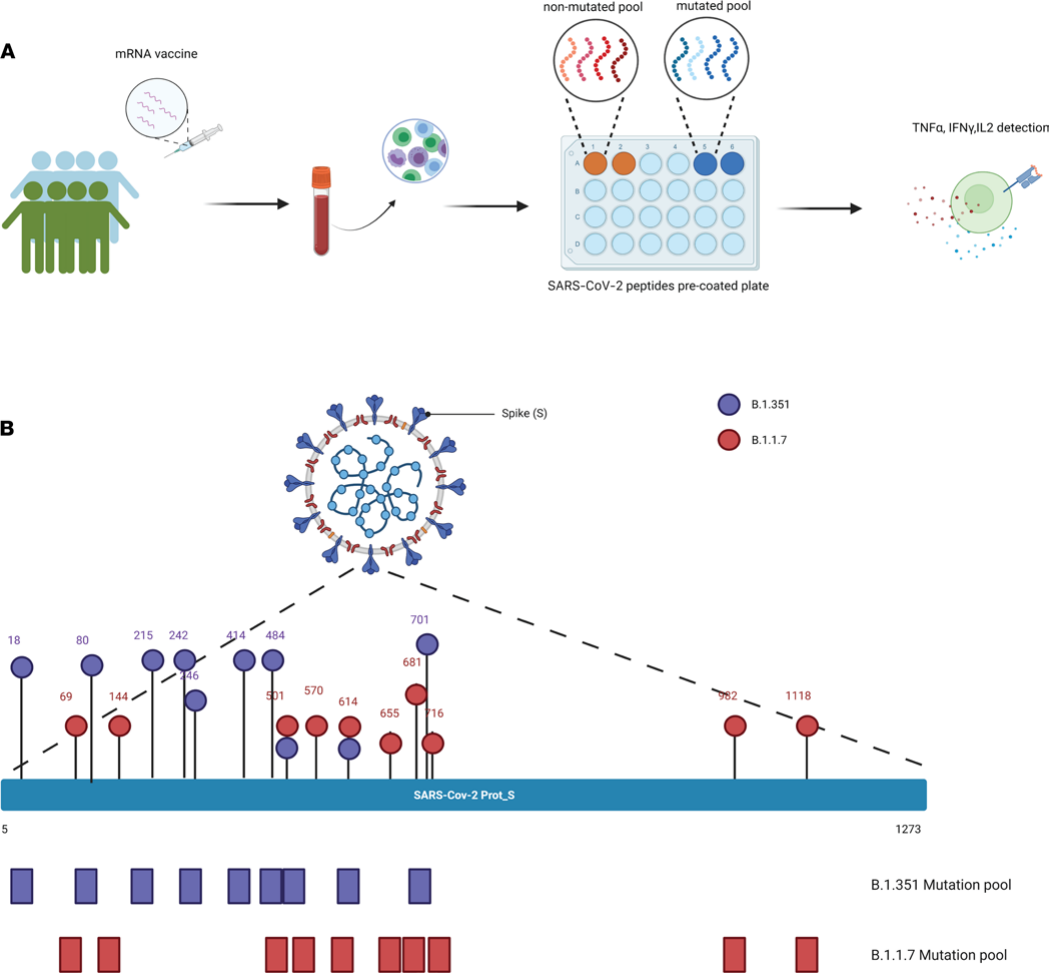
Study design. (**A**) A schematic illustration of the study design. (**B**) A schematic representation for SARS-CoV-2 peptide pools utilized in the study.

**Figure 2:**
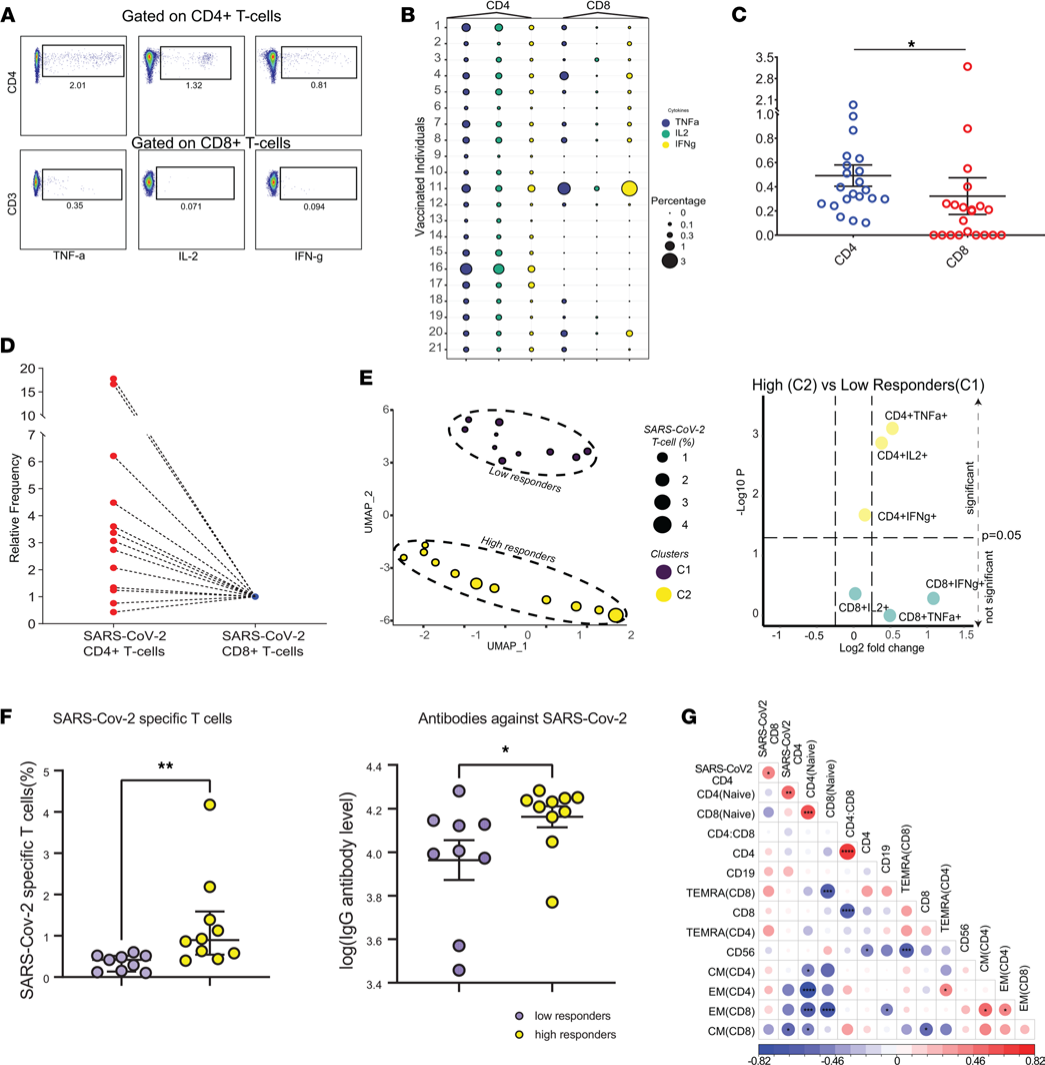
SARS-CoV-2 vaccination induces a CD4^+^ T cell–dominant immune response. (**A**) Representative FACS plots summarize cytokine production (TNF-α, IL-2, and IFN-γ) from CD4^+^ and CD8^+^ T cells in 1 of 21 donors. (**B**) The bubble plot shows the percentage of TNF-α–, IL-2–, and IFN-γ–secreting CD4^+^ and CD8^+^ T cells in 21 vaccinated individuals. The colors indicate cytokine and the size represents cytokine percentage per CD4 or CD8. (**C**) The bar plot shows the frequency of SARS-CoV-2–specific CD4^+^ and CD8^+^ T cells. Error bars represent mean ± SEM. (*P < 0.05 by Wilcoxon paired *t* test, *n* = 21.) (**D**) The relative percentages of SARS-CoV-2–specific CD4^+^ and CD8^+^ T cells in 13 individuals having detectable SARS-CoV-2–specific CD8^+^ T cells. (**E**) UMAP dimension and clusters identify differential response to vaccination (left). Volcano plot (right) shows the differentially expressed cytokines between C1 and C2 (Mann-Whitney *U* test, *n* = 21). (**F**) Plots show the comparison of SARS-CoV-2–specific T cells and IgG antibody level between low and high responders (*P < 0.05, **P < 0.01, unpaired *t* test, *n* = 21). Plots show the frequencies of SARS-CoV-2–specific T cells (CD4^+^ and CD8^+^) and the levels of S-protein–specific IgG. (**G**) The Spearman’s correlation matrices of immune features extracted from 21 vaccinated individuals (*P < 0.05, **P < 0.01, ***P < 0.001,****P < 0.0001, Wilcoxon paired *t* test, *n* = 21).

A total of 21 healthy donors with no history of prior COVID-19 infection and who received SARS-CoV-2 vaccines gave consented and were enrolled in this study. Twenty participants received mRNA-based vaccines while 1 participant received the Ad26.COV2.S vaccine (Johnson & Johnson/Janssen). The 20 donors received 2 doses of COVID-19 mRNA vaccine, and the peripheral blood samples (PB) were collected after the second dose of immunization. We then stimulated the PB mononuclear cells (PBMCs) with SARS-CoV-2 S-protein peptides for 6 hours and assessed the frequencies of SARS-CoV-2–specific T cells by analyzing the secretion of TNF-α, IFN-γ, and IL-2 (Figure 1A). To quantify the response against the mutated regions of S-protein of Alpha and Beta variants in COVID-19 mRNA vaccine recipients, we also stimulated the PBMCs in parallel with 2 different SARS-CoV-2 peptide pools spanning only the mutated S-protein regions of Alpha and Beta variants (Figure 1B) and 2 reference peptide pools containing the S-protein regions of Alpha and Beta variants as controls.

### SARS-CoV-2 vaccination induces a CD4^+^ T cell–dominant immune response.

As we assessed the SARS-CoV-2–specific T cell immune landscape, we thought phenotypic traits and cytokine expression patterns could provide insights into longevity and persistence of SARS-CoV-2–specific T cells. Thus, we sought to assess the abundance and qualitative features of vaccine-induced SARS-CoV-2–specific T cells and collected PB samples from 21 vaccinated individuals. Demographic features of study participants are summarized in Supplemental Table 1; supplemental material available online with this article; https://doi.org/10.1172/jci.insight.156559DS1; information on race and ethnicity were not collected. The median time from the second vaccine dose to sample collection for flow cytometric analysis was 95.7 days (range 13 to 110 days). To infer the abundance of circulating SARS-CoV-2–specific T cells, we utilized multiparametric flow cytometry and assessed the production of multiple cytokines including IFN-γ, TNF-α, and IL-2 after challenging PBMCs from vaccinated individuals with peptide pools spanning the whole length of S-protein (Figure 2A and Supplemental Figure 1A). Notably, vaccine-induced SARS-CoV-2–specific adaptive T cell immune response was detectable in every vaccine recipient (Figure 2B), which is in line with the efficacy observed in clinical trials (1, 2). However, we observed that IFN-γ, TNF-α, and IL-2 production by vaccine-induced SARS-CoV-2–specific CD4^+^ and CD8^+^ T cells was variable across the 21 vaccinated individuals (Figure 2B), suggesting that vaccination may provide a differential level of protection against COVID-19 due to varied magnitude of vaccine-induced SARS-CoV-2–specific T cell response. To ascertain that S-protein–specific immune response was induced after SARS-CoV-2 vaccination, we utilized frozen PB samples collected from 17 heathy donors prior to the COVID-19 pandemic or before the first COVID-19 case was detected in the United States as control samples and assessed the cytokine production after coculturing the PBMCs with S-protein peptide pools. Indeed, we only detected a miniscule fraction of reactive CD8^+^ T cells in 3 individuals, and CD4^+^ T cells were not reactive against S-protein after coculture (Supplemental Figure 1, B and C). These findings indicate that SARS-CoV-2–specific CD4^+^ and CD8^+^ T cells in vaccinated individuals emerged after vaccination since SARS-CoV-2–specific CD4^+^ and CD8^+^ T cells were detectable in every vaccine recipient. These findings are also in line with previous studies where S-protein–reactive T cells were detected in only a small number of healthy recipients at significantly lower quantities compared with individuals recovered from COVID-19 (9, 10).

To infer the abundance of circulating vaccine-induced SARS-CoV-2–specific T cells, we next utilized a Boolean gating approach to identify SARS-CoV-2–specific T cells producing any cytokine (IFN-γ, TNF-α, and IL-2) combinations and quantified SARS-CoV-2–specific CD4^+^ and CD8^+^ T cells. We observed that vaccination induced CD4^+^ T cell–dominant, SARS-CoV-2–specific immune response. In 19 out of 21 (85.7%) vaccinated individuals, the frequencies of CD4^+^ SARS-CoV-2–specific T cells were higher than their CD8^+^ T cell counterparts. Remarkably, SARS-CoV-2 CD8^+^ T cells were not detected in 8 individuals (Figure 2, B and C), indicating that the breadth of SARS-CoV-2–specific T cell response varies among vaccinated individuals. Indeed, previous reports demonstrated that mainly CD4^+^ T cells elicit robust cytokine responses against S-protein peptide pool after rechallenge in convalescent individuals with COVID-19, suggesting that cytokine profiles against S-protein in both convalescent individuals with COVID-19 and vaccinated donors overlap (11) and S-protein may favor the generation of SARS-CoV-2–specific CD4^+^ T cells. Next, we sought to determine the relative abundance of circulating SARS-CoV-2–specific CD4^+^ or CD8^+^ T cells and normalized their frequencies per CD4/CD8 ratio (Supplemental Figure 2A) since CD4^+^ and CD8^+^ T cells’ frequencies varied among vaccinated individuals. The median frequency of circulating SARS-CoV-2–specific CD4^+^ T cells was 3-fold (range: 0.42–39.8) higher than CD8^+^ T cell counterparts (Figure 2, C and D, and Supplemental Figure 2A) in 13 individuals where vaccine-induced SARS-CoV-2–specific CD4^+^ and CD8^+^ T cells were detected. Altogether, these findings showed that vaccination primarily induced a CD4^+^ T cell–dominant SARS-CoV-2–specific immune response.

Vaccine-induced SARS-CoV-2 T cell frequencies and cytokine expression levels varied across 21 donors. To detect the differential response to vaccination, we performed unsupervised clustering using the proportions of CD4^+^ and CD8^+^ T cells secreting IL-2, IFN-γ, and TNF-α. Notably, this unbiased approach segregated the vaccinated individuals into 2 distinct subpopulations: low responders (cluster 1) and high responders (cluster 2) (Figure 2E). IL-2– and TNF-α–producing CD4^+^ T cells were differentially enriched in individuals with higher fractions of vaccine-induced SARS-CoV-2 T cells (Figure 2E and Supplemental Figure 2B). Altogether, this finding indicates that vaccination induces a cellular immune response spectrum that varies across individuals. This phenomenon could translate to differential protection against COVID-19, and individuals with lower response might be more susceptible to COVID-19 infection. Although the majority of vaccinated individuals with higher levels of SARS-CoV-2 T cells received mRNA-1273 (Moderna) vaccine (8/11) (Supplemental Figure 2C), we did not observe significant difference with regard to immune correlates between individuals who received mRNA-1273 (11/21) versus BNT162b2 (9/21) vaccines (Supplemental Figure 2, D–F).

We have found that SARS-CoV-2 vaccines mainly induced a CD4^+^ T cell–dominant immune response. CD4^+^ T cells play a pivotal role in orchestrating the adaptive immune response and are essential for antibody isotype switch (12). Since CD4^+^ T cell–related immune correlates were the main feature segregating high versus low responders, we assessed the levels of S-protein–specific IgG in vaccinated individuals and grouped individuals according to high and low cellular immune responses. The vaccinated individuals with higher levels of cellular immune response as well had higher levels of S-protein–specific IgG, indicating that individuals with higher fractions of SARS-CoV-2–specific T cells tended to have higher levels of S-protein–specific IgG (Figure 2F). Thus, this may translate to variable protection across vaccinated individuals.

Next, we sought to investigate the fractions of major immune subsets and frequencies of distinct T cell states, including naive, central memory (CM), effector memory (EM), and terminally differentiated effector memory (TEMRA) (Supplemental Figure 3A), and investigated the association between SARS-CoV-2–specific T cell abundances and distinct immune states. First, we observed that the frequencies of SARS-CoV-2-specific CD4^+^ and CD8^+^ T cells were correlated (Figure 2G), indicating that individuals with higher fractions of SARS-CoV-2–specific CD4^+^ T cells tended to have higher frequencies of SARS-CoV-2–specific CD8^+^ T cells. Notably, we also found that vaccinated individuals with a larger pool of naive CD4^+^ T cells tended to have higher frequencies of SARS-CoV-2–specific CD4^+^ T cells (Figure 2G and Supplemental Figure 3B). The antigen-inexperienced T cells with unique sets of TCRs recognizing the S peptides reside in naive T cell compartment and give rise to SARS-CoV-2–specific T cells, and the pool size of naive T cells in vaccinated individuals may impose boundaries on the size of SARS-CoV-2 T cell pools. Importantly, reduction in naive T cells and T cell repertoire contraction have been linked to poor response to vaccination (13, 14), and elderly individuals with low frequencies of naive T cell fractions tend to contract severe COVID-19 infection (14, 15), suggesting that a shrunken pool of naive CD4^+^ T cells may alter the kinetics and limit the size of SARS-CoV-2–specific CD4^+^ T cells in vaccinated individuals. We also observed an association between the emergence of SARS-CoV-2–specific CD8^+^ T cells and the size of TEMRA CD4^+^ and CD8^+^ T cell pools (Figure 2G), further suggesting that individuals with a more permissible immune microenvironment for T cell differentiation are more likely to have higher abundance of SARS-CoV-2–specific CD8^+^ T cells and CD4^+^ T cells induced after vaccination.

### Cytokine profiles of vaccine-induced SARS-CoV-2–specific T cells.

**Figure 3:**
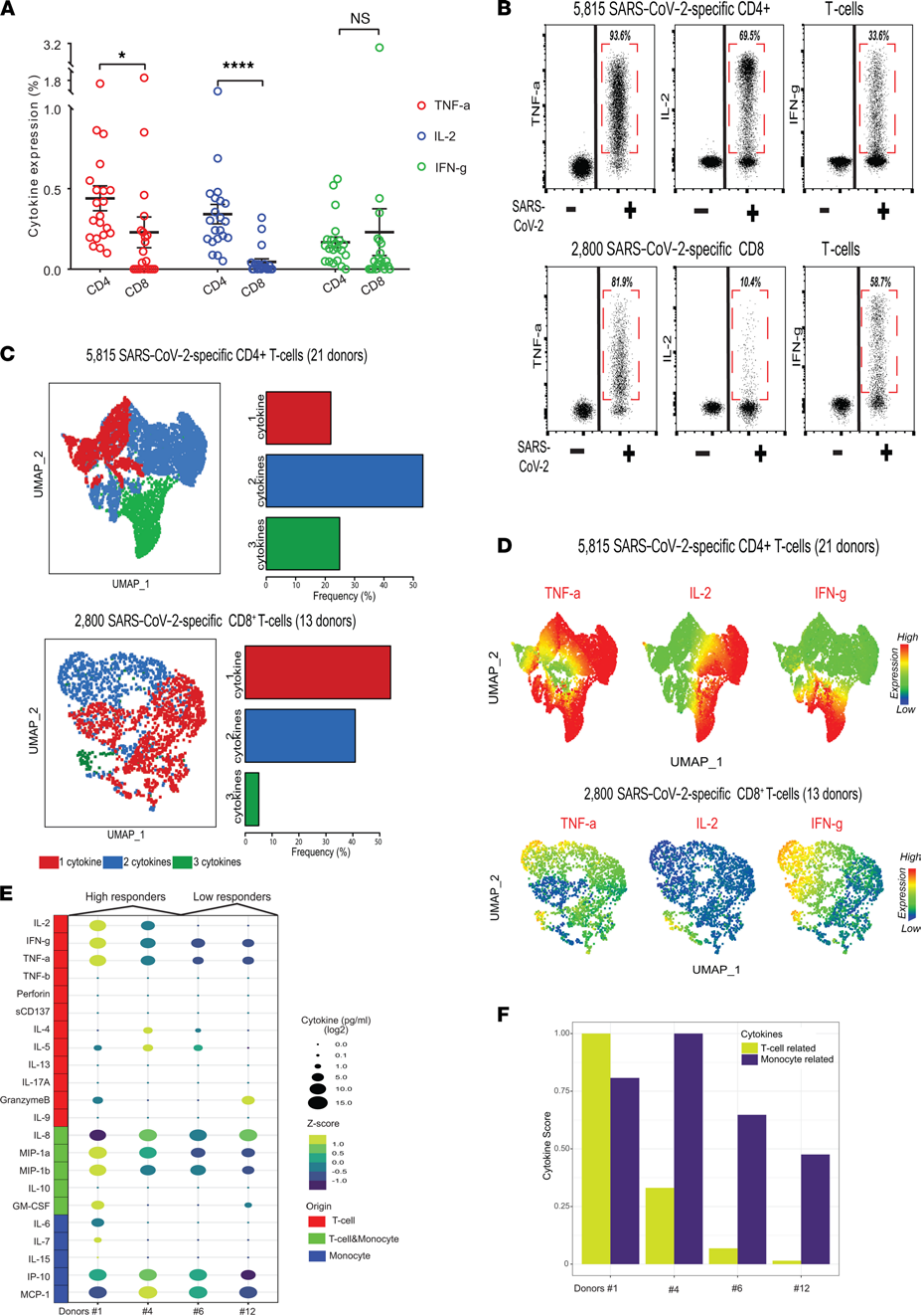
Cytokine profiles of vaccine-induced SARS-CoV-2–specific T cells. (**A**) Bar plots show the expression of TNF-α, IL-2, and IFN-γ in CD4^+^ T cells and CD8^+^ T cells. Error bars represent mean ± SEM (*P < 0.05, ****P < 0.0001, Wilcoxon paired *t* test, *n* = 21). (**B**) Dot plots summarize the expression of TNF-α, IL-2, and IFN-γ of SARS-CoV-2–specific CD4^+^ and CD8^+^ T cells from 21 vaccinated individuals. (**C**) The UMAP plots (left) show cytokine hierarchies of SARS-CoV-2–specific CD4^+^ (top) and CD8^+^ T cells (bottom). The bar plots (right) show the frequencies of SARS-CoV-2–specific CD4^+^ (top) and CD8^+^ (bottom) T cells secreting 1 (red), 2 (blue), and 3 (green) cytokines from 21 vaccinated individuals. (**D**) The UMAP plots show TNF-α, IL-2, and IFN-γ expression patterns of SARS-CoV-2–specific CD4^+^ (top) and CD8^+^ T cells (bottom). Color bars indicate the scaled expression levels. (**E**) The bubble plot shows the expression levels of 22 cytokines from 4 donors (2 high responders and 2 low responders). (**F**) Bar plot shows the T cell and monocyte cytokine scores from 4 donors. IP-10, IFN-γ–induced protein 10; MCP-1, monocyte chemoattractant protein-1; MIP-1a, macrophage inflammatory protein, UMAP, uniform manifold approximation and projection.

We utilized a multicolor flow cytometry approach to quantify the vaccine-induced SARS-CoV-2–specific T cells based on production of TNF-α, IL-2, or IFN-γ in our study. Since the ability to produce multiple cytokines by memory T cells is associated with superior recall functions (16, 17), we then sought to investigate cytokine profiles of vaccine-induced SARS-CoV-2–specific T cells. First, we determined the fractions of both CD4^+^ and CD8^+^ T cells producing TNF-α, IL-2, or IFN-γ (Figure 3A). Next, to attain a broader overview of the SARS-CoV-2–specific T cell landscape, we manually gated on and selected SARS-CoV-2–specific T cells, then merged all the events corresponding to either SARS-CoV-2–specific CD4^+^ (from 21 participants) or CD8^+^ (from 13 participants) T cells for the downstream analysis. This approach enabled in-parallel interrogation of SARS-CoV-2–specific CD4^+^ (a total of 5815 cells from 21 participants) and CD8^+^ (a total of 2800 cells from 13 participants) T cells (Figure 3A). Notably, we found TNF-α was the dominant cytokine produced by SARS-CoV-2 CD4^+^ (93.6%) and CD8^+^ (81.9%) T cells (Figure 3B). Moreover, 69.5% of the SARS-CoV-2-specific CD4^+^ T cells produced IL-2, a cytokine that could potentiate and enhance the persistence and longevity of SARS-CoV-2–specific T cells. However, only 33.6% of SARS-CoV-2–specific CD4^+^ T cells produced IFN-γ, the signature cytokine released from the Th1 cells. It is worth noting that T cells acquire IFN-γ production capability after several rounds of the cell cycle (18). These findings indicate that assays heavily relying on assessment of IFN-γ production, such as IFN-γ ELISPOT analysis (19), most likely underestimate the magnitude of SARS-CoV-2–specific T cell response in the context of COVID-19 vaccination. On the other hand, the frequencies of IFN-γ–producing, vaccine-induced, SARS-CoV-2–specific CD8^+^ T cells were comparable to those producing TNF-α whereas those producing IL-2 were scarce (Figure 3, A and C), illustrating contrasting cytokine expression profiles of SARS-CoV-2–specific CD8^+^ T cells in comparison with their CD4^+^ T cell counterparts.

Hierarchies in T cell cytokine expression profiles resemble the differentiation hierarchy observed in the T cell memory compartment. Less differentiated cells residing at the top of the hierarchy produce more IL-2, whereas more differentiated cells have a distinct cytokine expression pattern characterized by high levels of IL-4 and IFN-γ (20). TNF-α is generally produced by a multitude of CD4^+^ T cell subtypes representing a continuum of differentiation states (21) whereas highly differentiated CD4^+^ T cells lose the ability to produce TNF-α (22). Assessment of cytokine profiles could be of particular importance since the presence of polyfunctional memory T cells correlates with enhanced recall functions and superior protection (22). To determine the cytokine expression profile of SARS-CoV-2 T cells, we subjected SARS-CoV-2–specific T cells to UMAP dimension reduction and then utilized the Boolean gating approach to identify the proportions of SARS-CoV-2 CD4^+^ and CD8^+^ T cells producing single or multiple cytokines. The majority of SARS-CoV-2–specific CD4^+^ T cells produced at least 2 cytokines (Figure 3, C and D), and IL-2^+^TNF-α^+^ SARS-CoV-2–specific CD4^+^ T cells were the dominant subpopulation (Supplemental Figure 4A) of the COVID-19 immune landscape. On the contrary, SARS-CoV-2–specific CD8^+^ T cell functional profile in terms of cytokine production was more restricted, and SARS-CoV-2–specific CD8^+^ T cells mainly produced 1 or 2 cytokines (Figure 3C). TNF-α^+^ and TNF-α^+^IFN-γ^+^ SARS-CoV-2–specific CD8^+^ T cells were the most frequently encountered cell states in vaccinated individuals (Supplemental Figure 4B).

Using multiparametric flow cytometry, we identified TNF-α– and IL-2–producing CD4^+^ T cells as the hallmark of vaccine-induced SARS-CoV-2–specific T cell response. However, we were limited to interrogating the expression of 3 cytokines only per single cell using flow cytometry. Therefore, to gain a broader overview of cytokine secretion profiles and commitment states of SARS-CoV-2–specific T cells, we performed multiplexed ELISA using IsoPlexis’s CodePlex platform and assessed the secretion of 22 cytokines in culture supernatants after stimulating PBMCs from 4 vaccinated individuals (2 high responder donors versus 2 low responder donors) with S-protein peptide pools (Figure 3E). Remarkably, the levels of secreted IL-2, TNF-α, and IFN-γ in the bulk cytokines assay correlated with fractions of cytokine-secreting cells in the flow cytometry–based intracellular cytokine detection assay. Of note, IL-2 secretion was detected only in high responders, which was in agreement with the detection of high frequencies of IL-2–secreting CD4^+^ T cells using flow cytometry (Figure 2B). The assessment of the overall cytokine secretion profile revealed that SARS-CoV-2–specific T cells mainly produced Th1 cytokines including TNF-α and IFN-γ while they barely secreted Th2 (IL-4, IL-5, IL-10, and IL-13) and Th17-prone (IL-17A) cytokines, indicating that vaccine-induced SARS-CoV-2–specific CD4^+^ T cells mainly consisted of Th1 cells. Contrastingly, stimulation with S-protein peptide pool and crosstalk with T cells invariably induced high levels of monocytic cytokines including IFN-γ–induced protein 10 [IP-10], monocyte chemoattractant protein-1 [MCP-1], IL-8, macrophage inflammatory protein [MIP-1a], and MIP-1b, providing another aspect of the role of monocytes as a main source of cytokines in the context of COVID-19. To achieve an overall comparative assessment of T cell– and monocyte-related cytokines in high versus low responders, we generated T cell and monocyte cytokine scores (see Methods) derived by summation of T cell– and monocyte-related cytokines, respectively. As expected, T cell cytokine scores in high responders (donors #1 and #4) were higher, reflecting the higher abundance of vaccine-induced SARS-CoV-2 T cells and a more diverse secreted cytokine repertoire, as the results displayed that T cell–related cytokines were expressed significantly higher in high responders (Figure 3F).

### Phenotypic and evolutionary traits of vaccine-induced SARS-CoV-2–specific T cells.

**Figure 4:**
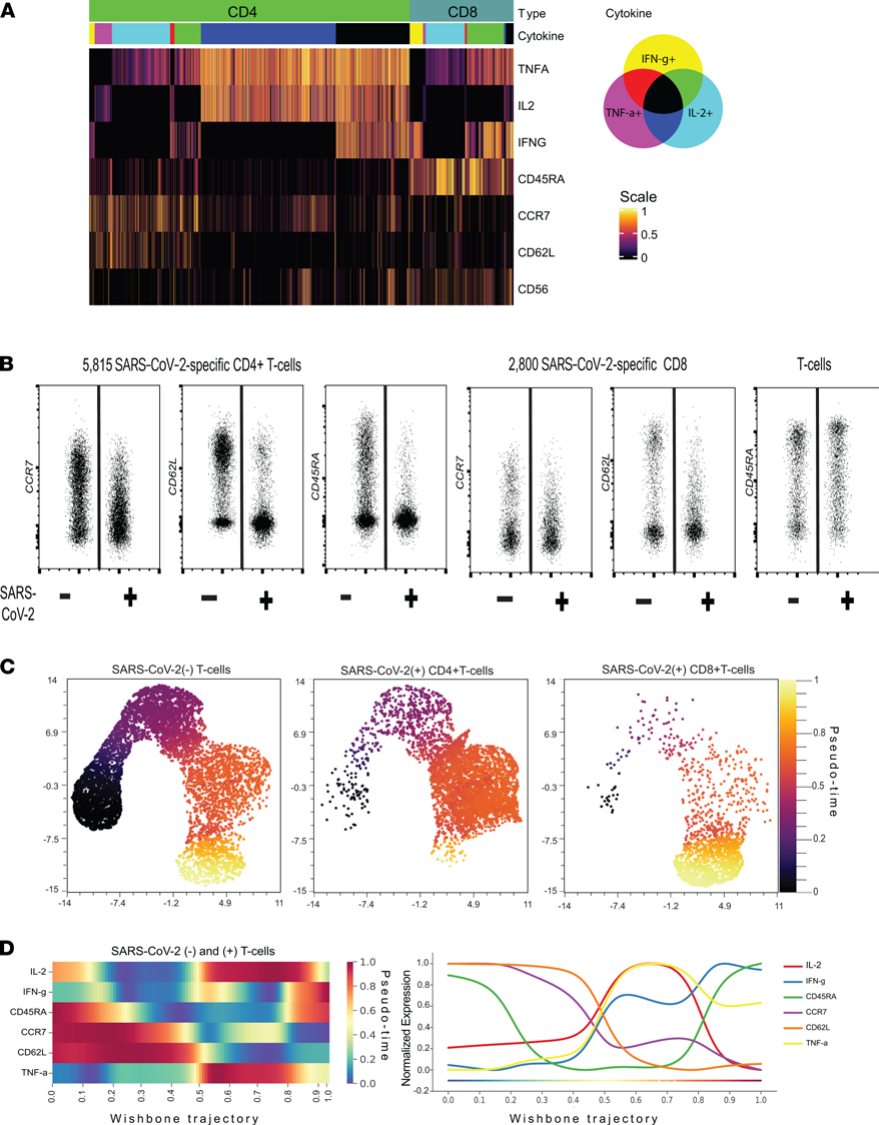
Phenotypic and evolutionary traits of vaccine-induced SARS-CoV-2–specific T cells. (**A**) Heatmap shows the expression of cytokines and differentiation markers of SARS-CoV-2–specific CD4^+^ and CD8^+^ T cells. The color bar indicates subsets producing different combinations of cytokines, and the scale bar shows the scaled expression level. (**B**) Dot plot shows the expression of CCR7, CD62L, and CD45RA in concatenated SARS-CoV-2–specific CD4^+^ (left) and CD8^+^ (right) T cells from 21 vaccinated individuals. (**C**) SARS-CoV-2–specific CD4^+^ (middle) and CD8^+^ (right) T cells and equal number of SARS-CoV-2^–^ T cells (left) were pooled and subjected to UMAP dimension reduction, and differentiation state of each single cell was inferred through pseudotime analysis. Pseudotime values are shown for each single cell. (**D**) Trend plots (left) and lines (right) show the expression levels of differentiation markers and cytokines along the pseudotime. Color scale indicates the scaled expression levels.

Next, we sought to assess the phenotypic landscape of SARS-CoV-2–specific T cells in vaccinated individuals and interrogate whether cytokine expression patterns of SARS-CoV-2–specific CD4^+^ and CD8^+^ T cells reflect their phenotypic traits or are associated with distinct phenotypic profiles. Despite producing a high level of IL-2, the majority of SARS-CoV-2–specific CD4^+^ T cells had an EM phenotype (CCR7^–^CD62L^–^CD45RA^–^). Notably, a fraction of SARS-CoV-2 CD4^+^ T cells exhibited a CM phenotype (CCR7^+^CD45RA^–^). In contrast, the majority of SARS-CoV-2–specific CD8^+^ T cells displayed a TEMRA phenotype (CCR7^–^CD62L^–^CD45RA^+^) (Figure 4A). Compared with SARS-CoV-2^–^ T cells, a fraction of SARS-CoV-2–specific CD4^+^ T cells expressed low/moderate levels of CCR7 (Figure 4B), which revealed the induction of distinct CD4^+^ T cell differentiation hierarchies after COVID-19 vaccination. It is well known that IL-2 is mostly produced by less differentiated CD4^+^ T cells, while IL-2–producing SARS-CoV-2–specific CD4^+^ T cells were almost exclusively confined to the EM T cell compartment (Supplemental Figure 5). SARS-CoV-2–specific CD8^+^ T cells exhibited a contrasting phenotypic profile compared with SARS-CoV-2–specific CD4^+^ T cells and contained a mixture of effector (CD45RA^–^CCR7^–^CD62L^–^) and terminally differentiated cells (CD45RA^+^CCR7^–^CD62L^–^) (Figure 4, A and B). Taken together, SARS-CoV-2 CD4^+^ T cells had a diverse cytokine expression profile, and the majority of them produced IL-2 despite having an EM phenotype. Indeed, the overall proteomic profiles of SARS-CoV-2–specific CD4^+^ T cells resembled long-lived polyfunctional T cells with distinct phenotypic features induced by vaccinia virus (23). Whether SARS-CoV-2–specific CD8^+^ T cells are long-lived, although they exhibit a more differentiated phenotypic profile compared with their CD4^+^ T cell counterparts, remains to be determined in follow-up studies.

We then performed pseudotime analysis to gain insights into differentiation states of SARS-CoV-2^+^ CD4^+^ and CD8^+^ T cells and constructed single-cell differentiation trajectories. First, we projected SARS-CoV-2^–^ T cells and SARS-CoV-2^+^ CD4^+^ and CD8^+^ T cells on 2-dimensional UMAP plots and inferred the pseudotime for each single cell to resolve single-cell pseudotime diversity and identify cellular abundances with distinct differentiation states across SARS-CoV-2^–^ and SARS-CoV-2^+^ T cell compartments (Figure 4C and Supplemental Figure 6). The SARS-CoV-2^–^ T cell compartment harbored a diverse continuum of T cell states while SARS-CoV-2 CD4^+^ T cells preserved a comparable differentiation continuum and contained multiple hierarchically organized differentiated T cell states, as shown by inferred pseudotime values (Figure 4C). Conversely, SARS-CoV-2^+^ CD8^+^ T cells exhibited a more restricted repertoire and were mostly situated at the end of the differentiation trajectory, corresponding to the terminal differentiation state characterized by CCR7^–^CD62L^–^CD45RA^+^. These findings suggest hierarchically organized, vaccine-induced SARS-CoV-2^+^ CD4^+^ T cell states could provide long-lasting protection and long-lived memory response since they are inherited with superior potential for proliferation and thus persistence. However, the association between differentiation state and diversity remains to be determined in future studies.

To infer the temporal acquisition of cytokine production by SARS-CoV-2 T cells along the T cell differentiation continuum, we mapped the dynamic relationship between the emergence of SARS-CoV-2 T cells and assessed the expression variance of cytokines and T cell differentiation events along the trajectory. This approach allowed us to time the key biological events and dissect the association between SARS-CoV-2 T cell differentiation hierarchies and cytokine expression profiles. We discovered 2 key events along the SARS-CoV-2 T cell differentiation continuum. First, the acquisition of high levels of TNF-α and IL-2 production capability coincided with overcoming differentiation checkpoints and emergence of SARS-CoV-2 T cells with an EM phenotype, such as T cells with downregulation of CCR7 and CD45RA (Figure 4D). Second, the progressive T cell differentiation was associated with a decline in the TNF-α and IL-2 output per single cell and induction of IFN-γ secretion, which coincided with reexpression of CD45RA. Overall, the single-cell trajectory analysis uncovered the association between cytokine expression profiles and SARS-CoV-2 T cell differentiation hierarchies. Indeed, it is a biologically interesting phenomenon that SARS-CoV-2 T cells, particularly CD4^+^ T cells, acquire the ability to produce IL-2 upon differentiating into EM T cells, though IL-2 is mainly produced by less differentiated T cells (24).

### Vaccine-induced SARS-CoV-2–specific T cells mainly recognize the non-mutated regions of S-proteins of Alpha and Beta VOCs.

**Figure 5:**
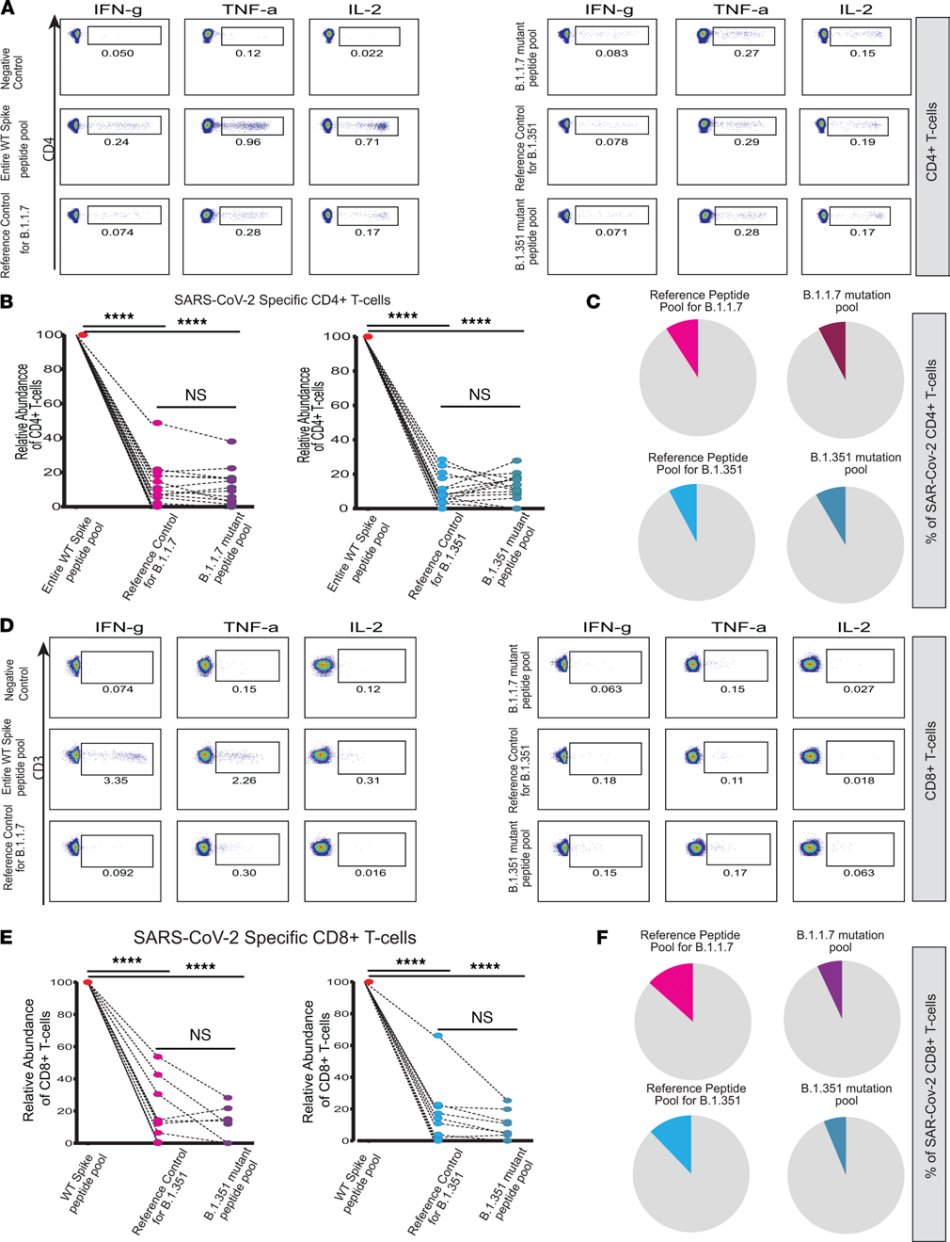
Vaccine-induced SARS-CoV-2–specific T cells mainly recognize the non-mutated region of S-proteins of Alpha and Beta VOCs. Representative FACS plots showing the expression of TNF-α, IL-2, and IFN-γ in CD4^+^ T (**A**) and CD8^+^ (**D**) following the stimulation with peptide pool spanning the entire length of WT S-protein and reference control and mutant peptide pools for B.1.1.7 (left) and B.1.351 (right), respectively. The plots show the relative abundance of SARS-CoV-2–specific CD4^+^ (**B**) and CD8^+^ (**E**) T cells following the stimulation with WT S-protein and reference control and mutant peptide pools of B.1.1.7 (left) and B.1.351 (right). The pie charts show the cumulative results for relative frequencies of SARS-CoV-2–specific CD4^+^ (**C**) and CD8^+^ (**F**) T cells reactive against reference controls and mutated peptides in **B** and **E** (****P < 0.0001, Wilcoxon paired *t* test, *n* = 21).

We hypothesized vaccine-induced T cells could still provide protection against VOCs since T cells recognize small peptides paired with HLA molecules and could still react against both mutated and non-mutated regions of S-protein presented through HLA molecules to CD4^+^ and CD8^+^ T cells. To test this hypothesis, we cocultured PBMCs from vaccinated individuals with the peptide libraries shown in Figure 1B: peptide libraries covering 1) the entire length of WT S-protein, 2) the mutated regions of S-protein of Alpha and Beta variants, and 3) homologous peptides of Wuhan sequence to serve as reference controls for the mutated regions of Alpha and Beta variant S-proteins. This gave us the opportunity to assess the proportion of SARS-CoV-2–specific T cells recognizing regions of WT S-protein homologous to Alpha and Beta variant S-protein mutation sites and test whether SARS-CoV-2–specific T cells recognizing WT homologous regions could also initiate an immune response against mutated peptides. First, we sought to quantify CD4^+^ T cell responses directed to WT homologous regions by comparing the abundance of CD4^+^ T cells reactive against S-protein homologous regions to mutated sites versus peptide libraries covering the entire length of WT S-protein. Strikingly, we found that the majority of SARS-CoV-2–specific CD4^+^ T cells’ recognition sites were situated outside the mutated S-protein regions of Alpha and Beta variants (Figure 5, A and B). Notably, we observed that only an average of 9.2% (reference control for Alpha) and 7.8% (reference control for Beta) of total SARS-CoV-2–specific CD4^+^ T cells were reactive against WT S-protein regions corresponding to the mutated sites of S-proteins of Alpha and Beta variants (Figure 5B). These findings unequivocally indicate that vaccine-induced SARS-CoV-2–specific CD4^+^ T cells could still confer protection against Alpha and Beta variants despite high burden of genetic alterations observed across the entire S-gene (25) since SARS-CoV-2–specific CD4^+^ T cells can efficiently mount an immune response through recognition of non-mutated amino acid sequences presented through HLA class II molecules. Next, we investigated whether SARS-CoV-2–specific CD4^+^ T cells could recognize and react against mutated sites of S-protein of Alpha and Beta variants and compared the fractions of CD4^+^ T cells reactive against peptide libraries covering only the mutated sites of S-proteins of Alpha and Beta variants versus their corresponding reference controls. Notably, SARS-CoV-2 CD4^+^ T cells showed no difference in the response to mutant peptide pool versus reference control peptide pool, revealing that SARS-CoV-2 CD4^+^ T cells were able to recognize the mutated regions across the S-proteins of both Alpha and Beta variants (Figure 5, A–C).

Previously, we observed that vaccination induced a sizable expansion of SARS-CoV-2–specific CD8^+^ T cells in a subgroup but not all of the vaccinated individuals. This gave us the opportunity to investigate whether SARS-CoV-2–specific CD8^+^ T cells can also recognize the mutant sites on S-proteins of Alpha and Beta variants in vaccinated individuals. Notably, the response patterns of SARS-CoV-2–specific CD8^+^ T cells to the mutated sites of Alpha and Beta variant S-proteins and reference controls were variable. In 6 vaccinated individuals, SARS-CoV-2 CD8^+^ T cells recognized only the non-mutated regions of the Alpha and Beta variant S-proteins, suggesting that SARS-CoV-2 CD8^+^ T cell response could be more restricted compared with SARS-CoV-2 CD4^+^ T cell response (Figure 5D). Furthermore, we observed reduced reactivity in SARS-CoV-2 CD8^+^ T cells against mutant sites of Alpha and Beta S-protein (Figure 5, E and F) in 4 individuals out of 7 individuals where SARS-CoV-2 CD8^+^ T cell response against WT homologous sites was detectable. This suggests that emerging mutations could compromise SARS-CoV-2 CD8^+^ T cell response directed against mutated regions while SARS-CoV-2 CD4^+^ T cell response against mutant regions was comparably unaltered. Together, our results showed that vaccine-induced SARS-CoV-2–specific CD4^+^ and CD8^+^ T cells could confer protection against VOCs mostly through recognition and reactivity against non-mutated regions of S-protein. Moreover, vaccine-induced SARS-CoV-2–specific CD4^+^ and CD8^+^ T cells also recognized mutated peptides, although we observed a partial reduction of CD8^+^ T cell response in a subset of individuals. Overall, these findings provide another valuable aspect of how vaccination could provide continued protection against emerging VOCs.

### Vaccination may induce durable SARS-CoV-2–specific cellular immunity.

We next sought to investigate the durability of vaccine-induced SARS-CoV-2–specific cellular immune response by assessing the abundance of SARS-CoV-2–specific T cells in serially collected samples from 7 individuals (3 high vs. 4 low responders) where we had available samples collected at 2 time points after vaccination. The median time from the second vaccine dose to the collection of first and second batches of samples was 106 days and 182 days, respectively. First, we compared the frequencies of vaccine-induced SARS-CoV-2–specific CD4^+^ and CD8^+^ T cells in samples collected 76 days apart. Remarkably, the abundance of circulating SARS-CoV-2–specific CD4^+^ and CD8^+^ T cells was comparable and there was no significant difference (Supplemental Figure 7A). Next, we assessed fractions of SARS-CoV-2–specific CD4^+^ T cells producing IL-2, TNF-α, and IFN-γ (Supplemental Figure 7B). Importantly, SARS-CoV-2–specific CD4^+^ T cells preserved IL-2 production, a cytokine that contributes persistence and proliferation. Next, we clustered CD4^+^ and CD8^+^ T cells by calculating fractions of cytokine production. This allowed us to achieve a broad assessment of vaccine-induced cellular immune landscape and to longitudinally track the immune dynamics. UMAP analysis revealed 2 distinct patterns (Supplemental Figure 7C). First, individuals with high and low immune response correlates clustered together at early and late time points, and the segregation was mainly driven by differential abundance of cytokine-secreting CD8^+^ and CD4^+^ SARS-CoV-2–specific T cells (Supplemental Figure 7D). Second, immune features assessed at early and late time points, either from high or low responders, were situated in close proximity on the high-dimensional plane, suggesting that the SARS-CoV-2–specific cellular immune landscape assessed at 2 time points was stable. Importantly, we did not observe a major change in cellular immune response status. High and low responders preserved the cellular immune features across 2 time points. Last, we performed a differential expression analysis to compare the frequencies of SARS-CoV-2–specific CD4^+^ and CD8^+^ T cells as well as cytokine secretion in response to stimulation and did not detect any significant changes of immune correlates assessed at early and late time points (Supplemental Figure 7E). Altogether, these findings indicate vaccination could induce a durable, stable, adaptive cellular immune response. However, future studies with longer follow-up and higher number of participants are warranted to shed light on the longevity of vaccine-induced SARS-CoV-2–specific cellular immunity.

## Discussion

Vaccines are desired to induce a long-lived adaptive immune response conferring durable protection. Whether COVID-19 vaccines can induce a durable and long-lived adaptive immune response is a major public health concern. A growing body of evidence reveals that COVID-19 vaccines are highly efficacious in preventing COVID-19 infections (2). Global emergence and rapid spread of VOCs have propelled interest to reveal the extent of protection that vaccine-induced SARS-CoV-2–specific adaptive immunity could provide against VOCs harboring mutant S-proteins. Although real-world experience and clinical studies revealed that vaccines, particularly, mRNA-based vaccines, are highly effective in preventing symptomatic COVID-19 infections, protection achieved through vaccination is not fail-safe (26). Breakthrough infections have been reported in a small number of vaccinated individuals, who tended to have lower neutralizing antibody concentrations (27, 28). However, the levels of SARS-CoV-2 S-protein–specific IgGs only partially correlate with risk of infection (29). While the anti–S-protein IgG and IgA play a role in prevention of SARS-CoV-2 infection by neutralization and prevention of cellular entry of SARS-CoV-2, antiviral T cell immunity can prevent viral replication in cells, eliminate the infected cells, and thus limit the disease severity. Therefore, in-parallel assessment of vaccine-induced SARS-CoV-2–specific humoral and cellular immune response will delineate vaccine-induced SARS-CoV-2–specific immune landscape and expedite the discovery of immune correlates of protection against breakthrough infections.

The risk and incidence of breakthrough infections could be higher than previously anticipated. A recent study demonstrated that breakthrough infection rate in health care workers was 2.6% and the Alpha variant, the predominant circulating VOC at the time, was responsible for the vast majority of breakthrough infections (27). This suggests that vaccination may induce a variable spectrum of adaptive immune response and individuals with lower magnitude and breadth SARS-CoV-2–specific immune response may potentially be at a higher risk of contracting breakthrough infections. Our analysis of immune repertoire of vaccinated individuals revealed a heterogenous vaccine-induced immune response landscape, and SARS-CoV-2–specific CD4^+^ and CD8^+^ T cell frequencies varied among vaccinated individuals.

Previous studies indicate that preexisting antiviral immunity against various coronaviruses could provide crossprotection against COVID-19 infection (30). However, preexisting T cells detectable in PB of unexposed individuals recognized mainly SARS-CoV-2 proteins other than S-protein, and the reactivity against S-protein peptide pools was negligible (9). In another study, 1 out 10 unexposed healthy controls had response against S-protein at comparably much lower quantities compared with S-protein–reactive T cells in individuals recovered from COVID-19 infection (10).

We also found that S-protein–specific IgG levels varied across vaccinated individuals and were higher in individuals with a higher magnitude of SARS-CoV-2–specific cellular immune response. CD4^+^ T cells are the master regulators of adaptive immune response, and, therefore, it is reasonable to assume that higher frequencies of SARS-CoV-2–specific CD4^+^ T cells could facilitate and propagate antibody isotype switching and could be associated with higher levels of S-protein–specific IgG levels. Whether the difference in the breadth and magnitude of vaccine-induced SARS-CoV-2–specific T cell responses and humoral immunity could translate into differential protection against various VOCs is unknown. It is reasonable to assume that vaccinated individuals situated at the lower end of the adaptive immune response spectrum are more susceptible to contracting COVID-19 infection. However, the association between the SARS-CoV-2–specific cellular and humoral immune landscape and protection against COVID-19 infection remains to be further explored in future studies. In-parallel assessment of humoral and cellular immune landscapes may provide a more in-depth understanding of the level of protection against COVID-19 infection in vaccinated individuals.

SARS-CoV-2 vaccines arm adaptive immunity by inducing memory B cells and plasmablasts expressing and secreting specific antibodies, respectively, and SARS-CoV-2–specific CD4^+^ and CD8^+^ T cells recognize cleaved fragments of S-proteins presented on MHC class I and II molecules. Vaccine-induced SARS-CoV-2–specific antibodies persist through 6 months postvaccination, and whether vaccine-induced neutralizing antibodies can persist beyond 6 months is yet to be determined (31). Moreover, Beta, Alpha, and Gamma variants can evade neutralizing antibodies owing to mutations in receptor binding and N-terminal domains (32, 33). Therefore, vaccine-induced SARS-CoV-2–specific cellular immunity could serve as the essential arm in conferring continued protection against various current and emerging VOCs where humoral immunity could be more likely to be compromised. Therefore, assessment of the magnitude and breadth of vaccine-induced SARS-CoV-2–specific T cell response along with humoral immune response could provide insights into whether SARS-CoV-2–specific adaptive immune response could provide protection against COVID-19 infection driven by emerging VOCs. Importantly, we found that vaccination elicited mainly a CD4^+^ T cell–dominant immune response in the vast majority of vaccinated individuals and 8 individuals lacked SARS-CoV-2–specific CD8^+^ T cell response. Moreover, the abundance of SARS-CoV-2–specific CD4^+^ T cells was significantly higher than their CD8^+^ T cell counterparts in individuals with detectable SARS-CoV-2–specific CD8^+^ T cells. Vaccinated individuals lacking or with low abundance of SARS-CoV-2–specific CD8^+^ T cells may be more vulnerable to VOC-associated COVID-19 infections. Various VOCs can readily evade humoral immune response (33) and infect epithelial cells of the airway tract, which express high levels of angiotensin-converting enzyme 2 receptors and lack HLA class II molecules. In this scenario, high abundance of SARS-CoV-2–specific CD4^+^ T cells cannot provide protection and eliminate infected epithelial cells since only SARS-CoV-2 CD8^+^ T cells can recognize and eliminate infected cells expressing MHC class I molecules. On the other hand, SARS-CoV-2–specific CD4^+^ T cells can initiate a SARS-CoV-2–specific immune response only when MHC class II–expressing cells, namely B cells, monocytes, and dendritic cells, are infected and present S-protein peptides through MHC class II molecules. Therefore, infected epithelial cells could serve as a “safe reservoir” for replication in vaccinated individuals lacking a sizable pool of SARS-CoV-2–specific CD8^+^ T cells. SARS-CoV-2 can also infect monocytes and dendritic cells (34), resulting in the release of a plethora of cytokines causing cytokine release syndrome (CRS). Vaccine-induced, relatively more abundant, SARS-CoV-2–specific CD4^+^ T cells can eliminate infected monocytes, immune cells implicated in CRS and systemic inflammatory syndrome, and halt disease progression and prevent severe infection. This could be the underlying mechanism to explain why vaccinated individuals can still contract SARS-CoV-2 infections, yet do not develop severe disease, since SARS-CoV-2 CD4^+^ T cells, consistently induced after vaccination, could serve as the late-acting gatekeepers preventing the uncontrolled propagation of SARS-CoV-2 infection. Vaccine-induced SARS-CoV-2–specific CD4^+^ T cells could pose as the main line of defense against COVID-19 in vaccinated individuals, since VOCs can evade neutralizing antibodies and SARS-CoV-2–specific CD8^+^ T cells are not invariably induced after vaccination at appreciable quantities. These assumptions are in agreement with real-world experience with SARS-CoV-2 vaccines utilizing different approaches to induce immunity mainly targeting S-protein, where SARS-CoV-2 vaccines may fail to prevent mild-to-moderate breakthrough infections at least in a subgroup of vaccinated individuals but be highly effective at reducing the risk of severe infections and hospitalizations (35, 36). Correlates of protection against VOC-driven COVID-19 infections are not fully established. However, integrated assessment of vaccine-induced humoral and cellular SARS-CoV-2–specific immunity could provide an additional layer of information to determine correlates associated with superior protection.

Whether SARS-CoV-2 vaccines induce long-lived SARS-CoV-2–specific T cells remains to be answered, and future studies with longer follow-up and assessing the cellular response years after vaccination could provide a clear answer. We reasoned that in-depth analysis of the response landscape in vaccinated individuals could provide insights into longevity of vaccine-induced SARS-CoV-2 T cells. To achieve a broad overview and in-depth analysis of the SARS-CoV-2–specific T cell landscape, we pooled all the SARS-CoV-2–specific T cells from 21 donors and performed single-cell analysis to identify correlates associated with longevity and persistence. Importantly, single-cell analysis revealed that phenotypic traits, cytokine profiles, and differentiation hierarchies of SARS-CoV-2 CD4^+^ T cells contrasted with those of SARS-CoV-2 CD8^+^ T cells. The majority of SARS-CoV-2 CD4^+^ T cells were differentiated T cells having an EM phenotype and situated at an earlier stage along the differentiation continuum in comparison with SARS-CoV-2 CD8^+^ T cells. Remarkably, the SARS-CoV-2 CD4^+^ T cell landscape harbored a diverse continuum of T cell states, and we also identified less differentiated SARS-CoV-2 CD4^+^ T cells situated at an earlier differentiation stage while SARS-CoV-2 CD8^+^ T cells had a less diverse landscape and were mostly composed of TEMRA cells. Whether a more diverse differentiation landscape and the presence of relatively less differentiated SARS-CoV-2 CD4^+^ T cells would translate into superior longevity remains to be determined. A recent report revealed that stem cell–like memory T cells, a unique T cell differentiation state highly associated with long-term T cell memory (37), were induced and detectable through 10 months in COVID-19 convalescent individuals (38). In our analysis, we also observed less abundant SARS-CoV-2 CD4^+^ T cells with naive-like T cell phenotype residing at the apex of cellular hierarchy, indicating that vaccination may also induce less differentiated SARS-CoV-2 CD4^+^ T cells with superior T cell memory potential. However, our flow cytometry panel did not include CD95, and therefore we could not ascertain the precise phenotype of less differentiated SARS-CoV-2 CD4^+^ T cells in our study.

In line with distinct hierarchical organizations of SARS-CoV-2 CD4^+^ versus CD8^+^ T cells, we discovered stark differences in cytokine expression patterns between SARS-CoV-2 CD4^+^ versus CD8^+^ T cells. Our study revealed SARS-CoV-2 CD4^+^ T cells were polyfunctional, and a high fraction of these cells produced IL-2, which could support long-term T cell memory response. Notably, IL-2 and TNF-α coproducing T cells were the dominant population within the SARS-CoV-2 immune landscape. The phenotypic features and cytokine expression profiles of SARS-CoV-2 CD4^+^ T cells resembled those of T cells induced in response to various vaccines and associated with long-term memory and superior protection (23, 39). Notably, polyfunctional cytokine profiles and distinct phenotypic traits of SARS-CoV-2 CD4^+^ T cells could be indicative of enhanced recall functions and longevity (22, 40). On the contrary, SARS-CoV-2 CD8^+^ T cells had a restricted cytokine expression profile, suggesting that they may not exert optimal recall functions and may undergo memory T cell decay. Our longitudinal assessment of vaccine-induced SARS-CoV-2 response in samples collected 3 months apart revealed SARS-CoV-2 immunity was preserved, and there was no evidence of significant decay in SARS-CoV-2 T cell response.

Our study has certain limitations since the adaptive immune response was assessed only in 21 vaccinated individuals. Although the number of participants in the study was relatively low, the unbiased clustering analysis was capable of delineating an adaptive immune response that varied in magnitude and identified 2 distinct groups with high versus low immune correlates. Whether our data-driven immune response stratification approach could translate to superior protection against COVID-19 remains to be determined in a larger cohort of participants with a longer follow-up. We did not collect PB samples from vaccinated individuals prior to vaccination, which could have ascertained that SARS-CoV-2–specific cellular and humoral immune response emerged after vaccination. However, to provide further evidence, we have utilized and assessed PB samples collected prior to the COVID-19 pandemic, and we observed that a very low abundance of S-protein–specific T cell response was detectable only in 2 individuals. These findings corroborated the conclusion that SARS-CoV-2–specific cellular immunity emerged after vaccination. As for the assessment of humoral immune response, we did not quantify the S-protein–specific IgM levels since mRNA vaccines induce weaker IgM antibody response compared with IgG response (41, 42) and the plasma samples were collected mainly 3 months after the second dose of vaccination. Only in 1 donor (donor 18) were both PBMCs and plasma samples collected early after the second dose of vaccination. However, we observed that the cytokine expression patterns in the donor sample collected early after the second vaccination (Figure 2B) were similar to cytokine expression patterns of samples collected at later time points with regard to the proportion and type of produced cytokines. Therefore, the data points from this sample were included in the concatenated data matrix to assess and delineate the SARS-CoV-2–specific T cell cytokine and proteomic hierarchies in an unbiased manner.

It is also worth noting that we have not utilized additional studies to investigate whether a higher magnitude of SARS-CoV-2–specific cellular and humoral immunity reflects superior functional attributes, including cytotoxicity, neutralization, and crossreactivity. Nevertheless, flow cytometry–based quantification of SARS-CoV-2–specific T cells is a functional assay where the production of a multitude of cytokines was assessed. High cytokine production could reflect superior cytotoxicity, and in vitro cytotoxicity assays using S-protein peptide pool–pulsed antigen-presenting cells could be utilized in future studies to reveal the association between cytokine production and cytotoxicity.

Ever-shifting mutation landscape of SARS-CoV-2 led to emergence of more transmissible novel VOCs, which could partially avoid humoral immunity and cause COVID-19 infection. We hypothesized that vaccine-induced SARS-CoV-2 T cells could still react against mutated S-protein and infected cells since vaccine-induced SARS-CoV-2 T cells could recognize non-mutated fragments of S-protein, which is composed of 1273 amino acids. MHC class I molecules generally display 8 to 10 aa–long peptides while MHC class II present peptides more than 11 aa (43). This indicates that vaccine-induced SARS-CoV-2 T cell response could recognize numerous distinct sites of S-protein. MHC class I– and II–restricted S-protein epitopes presented to CD8^+^ and CD4^+^ T cells, respectively, vary among vaccinated individuals depending on one’s overall HLA makeup, and T cells from different individuals could recognize completely non-overlapping sites of S-protein. Theoretically, overall vaccine-induced T cell response could be directed against non-mutated regions of S-protein and could provide unaltered protection against various VOCs. Indeed, we found that vaccine-induced SARS-CoV-2 T cell response was predominantly directed against non-mutated regions of B1.1.7 and B1.351 variants. Of note, we discovered that a small fraction of vaccine-induced SARS-CoV-2 T cells recognize mutated regions and found that SARS-CoV-2 T cell response, particularly CD8^+^ T cell response, specific for mutated regions was partially reduced in a subgroup of vaccinated individuals. Several studies reported that vaccine-induced SARS-CoV-2 T cells responded similarly to S-protein from WT and various VOCs by utilizing peptide libraries spanning the whole length of mutated S-proteins (44). Contrastingly, we used peptide libraries spanning only the mutated regions of S-proteins from various VOCs. This gave us the opportunity to delineate and quantify vaccine-induced SARS-CoV-2 T cell response directed against non-mutated versus mutated regions of B1.1.7 and B1.351 S-proteins. Indeed, we uncovered that the majority of SARS-CoV-2 T cell response was directed against non-mutated regions of VOC S-proteins, indicating that vaccine-induced SARS-CoV-2 T cell response could confer protection irrespective of mutation status. Notably, our study also provides the proof of concept that SARS-CoV-2 T cells could provide protection against emerging VOCs owing to their ability to recognize non-mutated regions of VOC S-proteins. In this study we uncovered potentially unique properties of vaccine-induced SARS-CoV-2 T cell immunity, distinct differentiation hierarchies, and cytokine expression patterns. Overall, the traits of SARS-CoV-2 T cells, particularly CD4^+^ T cells, indicate that vaccination may induce a long-lived T cell response and provide crossprotection against emerging novel VOCs.

## Methods

### Samples.

PB samples were collected from 21 fully vaccinated donors, and informed consent was obtained in accordance with The University of Texas MD Anderson Cancer Center Institutional Review Board (IRB) guidelines. PB samples from 17 healthy donors collected for prior, unrelated studies before the COVID-19 pandemic were used to assess preexisting T cell responses to SARS-CoV-2 (Supplemental Table 2). PBMCs were isolated by centrifuge at 400*g* for 20 minutes with the brake off at room temperature using lymphocyte separation medium (Lymphoprep, Axis Shield). PBMCs isolated from unexposed individuals prior to the advent of SARS-CoV-2 vaccines were cryopreserved in freezing media containing 10% dimethyl sulfoxide and 90% fetal bovine serum. Frozen PBMCs were thawed rapidly in 37°C water bath, and half-thawed samples were transferred to prewarmed culture media (90% RPMI 1640) containing 2 mM GlutaMAX TM-I (both from Gibco), 1% penicillin-streptomycin, and 10% fetal bovine serum (FBS). Freshly isolated or thawed PBMCs were seeded at a density of 5 × 10^5^/mL in 96-well, round-bottom plates (Corning) and incubated overnight prior to peptide stimulation.

### Peptides.

SARS-CoV-2 Prot_S, SARS-CoV-2 Prot_S1, and SARS-CoV-2 Prot_S^+^ peptide pools consisting of 15-mer overlapping peptides covering the whole-length WT S-protein, B.1.1.7. (Alpha) S-protein (mutated peptides and corresponding WT controls), and B.1.351 (Beta) S-protein (mutated peptides and corresponding WT controls) peptide pools were sourced from Miltenyi Biotec. The peptide pools were dissolved in sterile water at a stock solution concentration of 30 nmol/mL/peptide per manufacturer’s instructions. The final concentration for each peptide used for stimulation was 1 μg/mL. The PBMCs were suspended in cell culture medium (RPMI 1640 supplemented with 10% FBS, 1% l-glutamine, and 1% penicillin/streptomycin) and plated at 0.5 × 10^6^/well. Fresh PBMCs were stimulated with the peptide pools for 6 hours in the presence of brefeldin A (BioLegend) in 96-well, round-bottom plates as described previously (45, 46). Negative controls without peptide stimulation were also included for each donor sample. Negative control values were subtracted from cytokine values obtained after stimulating PBMCs with S-protein peptide pool. The negative values were set to 0. To quantify the fractions of SARS-CoV-2–specific CD4^+^ and CD8^+^ T cells secreting different cytokines, we gated on CD4^+^ and CD8^+^ and calculated the fractions of CD4^+^IL-2^+^, CD4^+^IFN-γ^+^, CD4^+^TNF-α^+^, CD8^+^IL-2^+^, CD8^+^IFN-γ^+^, and CD8^+^TNF-α^+^ cells.

### SARS-CoV-2 S-protein IgG detection.

To quantify the IgG levels against the trimeric full-length S-protein, we utilized human SARS-CoV-2 spike (trimer) (ELISA kit from Thermo Fisher Scientific) per manufacturer’s instructions. Matched plasma samples and PBMCs, used to quantify SARS-CoV-2–specific T cells as described above, were collected from vaccinated individuals at the same time. Plasma samples were frozen and stored at –80°C for long-term storage. Frozen plasma samples were thawed on ice, and 10 μL of plasma samples was used to quantify SARS-CoV-2 S-protein–specific IgG levels. In brief, ELISA plates coated with S-protein were washed twice, and 90 μL of assay buffer was added to each well. A total of 10 μL of plasma samples in replicate was added, and the samples were incubated for 30 minutes at 37°C. The wells were washed 3 times with wash buffer, followed by adding 100 μL HRP buffer and incubating 30 minutes at 37°C. The wells were washed and 100 μL substrate solution was added to each well. Then 100 μL of stop solution was added to each well. The absorbance at 450 nm was assessed.

### Flow cytometry.

After stimulation, cells were washed twice with cell staining buffer (0.5% bovine serum albumin/PBS) and incubated with 5 μL of human Fc receptor blocking solution (Trustain FcX, BioLegend) for 10 minutes at room temperature. Cells were then stained with LIVE/DEAD Fixable Aqua Dead Cell Stain Kit (Thermo Fisher Scientific) for 20 minutes at room temperature (RT) in the dark, and then cells were stained with an antibody mixture including anti-CD3–BUV395 (BD Biosciences, catalog 563548), anti-CCR7–FITC (BD Biosciences, catalog 561271), anti-CD4–BV785 (BioLegend, catalog 317442), anti-CD8–AF700 (BioLegend, catalog 300920), anti-CD45RA–BV711 (BioLegend, catalog 304138), anti-CD62L–PerCP-cy5.5 (BioLegend, catalog 304824), and anti-CD56–BV605 (BioLegend, catalog 362538) for 30 minutes at RT. Then cells were washed twice with cell staining buffer and fixed/permeabilized using BD Cytofix/Cytoperm solution for 30 minutes in dark at 4°C per manufacturer’s instructions. Cells were washed twice with perm/wash buffer, stained with antibodies directed against intracellular cytokines (anti–TNF-α–PE-Cy7, BioLegend catalog 502930; anti–IFN-γ–PE, BioLegend catalog 506507; and anti–IL-2–APC, BioLegend clone MQ1-17H12) for 30 minutes in the dark at 4°C. Finally, cells were washed in PBS before analysis on Cytek Aurora flow cytometer equipped with 5 lasers (Cytek Biosciences Inc). Initial flow cytometry data analysis was performed using FlowJo v.10.7.1 software (BD Biosciences). To quantify the fractions of SARS-CoV-2–specific CD4^+^ and CD8^+^ T cells secreting different cytokines, we gated on CD4^+^ and CD8^+^ and calculated the fractions of CD4^+^IL-2^+^, CD4^+^IFN-γ^+^, CD4^+^ TNF-α^+^, CD8^+^IL-2^+^, CD8^+^IFN-γ^+^, and CD8^+^ TNF-α^+^ cells.

### Cytokine profiling of donor PBMCs.

PBMCs from 4 donors (2 high responders and 2 low responders) were isolated and stimulated with WT SARS-CoV-2 S-protein peptide pools for 12 hours. After incubation, supernatants were collected and loaded to CodePlex chip (IsoPlexis) that allowed the assessment of 22 human cytokines per manufacturer’s instructions. Chips were read in IsoLight instrument and data were analyzed in Isospeak software.

### Boolean analysis.

A Boolean gating strategy implemented in FlowJo v.10.7.1 software was used to identify and quantify SARS-CoV-2 CD4^+^ and CD8^+^ T cells expressing various combinations of IL-2, TNF-α, and IFN-γ. First, we gated on singlets and excluded dead cells based on LIVE/DEAD aqua viability stain; determined the fractions of TNF-α, IFN-γ, and IL-2; and performed Boolean gating to determine the various cytokine combinations secreted by CD4^+^ and CD8^+^ T cells. SARS-CoV-2 CD4^+^ and CD8^+^ T cells with different cytokine combinations were concatenated to quantify SARS-CoV-2 CD4^+^ and CD8^+^ T cells for 21 individuals. To assess the overall SARS-CoV-2 T cell repertoire, FCS files containing SARS-CoV-2 CD4^+^ and CD8^+^ T cells were concatenated into 2 separate FCS files.

### Cytokine score.

T cell cytokine score was calculated by summing the levels of cytokines and soluble factors known to be produced by memory T cells (TNF-α, IL-2, IFN-γ, granzyme B, IL-4, IL-5, MIP-1a, MIP-1b, GM-CSF). Monocyte cytokine score was calculated by the summing the amounts of monocyte-associated cytokines (MCP-1, IP-10, IL-6, IL-7, MIP1a, MIP-1b, and IL-8).

### Dimension reduction.

We utilized the FlowJo UMAP plugin for dimension reduction and visualized the flow cytometry data in 2 dimensions. We gated on and selected SARS-CoV-2 CD4^+^ and CD8^+^ T cells from 21 donors and concatenated them into separate FCS files. Pooled SARS-CoV-2 CD4^+^ and CD8^+^ T cells were subjected to UMAP dimension reduction. To assess the cytokine hierarchies in Figure 3, C and D, we used IFN-γ, TNF-α, and IL-2 for dimension reduction. To infer the number of cytokines expressed per single cell, we subjected the pooled SARS-CoV-2 CD4^+^ and CD8^+^ T cells to Boolean gating analysis and partitioned the cells into cell subgroups producing different cytokine combinations. Cytokine secretion patterns were then projected on UMAP plots.

### Pseudotime inference.

We utilized Wishbone and Monocle3 to perform trajectory analysis and pseudotime inference for SARS-CoV-2 CD4^+^ and CD8^+^ T cells. To perform Wishbone, concatenated and exported FCS files containing events corresponding to SARS-CoV-2 CD4^+^ and CD8^+^ T cells were uploaded to Omiq.ai for Wishbone trajectory analysis. Expression values were transformed by arcsinh, and cells were subjected to UMAP dimension reduction to project the data in 2 dimensions. We then made diffusion maps to identify the major trend in the data as a part of trajectory analysis. We selected 3 informative diffusion components based on plotting the diffusion components versus algorithm-generated eigenvalues for trajectory analysis. We plotted the data in 2 dimensions using UMAP plots and projected the pseudotime values on UMAP plots to infer cellular relatedness along pseudotime.

Monocle3 (47) was used to perform trajectory and pseudotime inference for CD4^+^ and CD8^+^ T cells, respectively, using biexponential transformed values of CD45RA, CD62L, and CCR7. It embeds the data into UMAP space, selects “landmark” cells by k-means clustering, and infers a tree of those cells using SimplePPT algorithm. Pseudotime is inferred by calculating the geodesic distance to the root (i.e., SARS-CoV-2–negative CD4^+^ and CD8^+^ T cells). To compare the differentiation progress of CD4^+^ and CD8^+^ T cells, a consensus UMAP is then calculated for the union of both cell types and overlaid with the inferred pseudotime.

### Heatmap.

To account for background noise in flow cytometry, we examined the distribution of the level of each marker and selected a cutoff value using FlowJo. A level smaller than the threshold is considered not detected and shown as 0 in the heatmap. To visualize the highly skewed distribution, levels higher than the threshold are displayed by their ranks. Cells are grouped by cell types and expressed cytokines.

### Clustering of vaccinated individuals.

Seurat was used to cluster the 21 patients based on the abundance of TNF-α–, IFN-γ–, and IL-2–producing CD8^+^ and CD4^+^ T cells. UMAP embedding based on 4 principal components was used to separate 2 groups of vaccinated individuals.

### Statistics.

Statistical analyses were performed using Prism version 7.0 (GraphPad Software Inc.) and using R software version 3.6.3. The statistical differences between matched groups were compared using a paired *t* test using GraphPad Software. To compare unpaired groups, we used Mann-Whitney *U* test. The Spearman rank correlation was used to assess correlation. (Statistical significance was set at *P* < 0.05: *P < 0.05; **P < 0.01; ***P < 0.001; ****P < 0.0001.)

### Study approval.

IRB approval was obtained from The University of Texas MD Anderson Cancer Center. Written informed consent was obtained from all participants.

## Author contributions

LL, MM, and SP conceptualized the study. LL, MM, JL, and MB performed experiments. LL, MM, SL, and MEA analyzed the data and generated the figures. KC, MA, and CRF interpreted data and reviewed the manuscript. LL, MM, SL, and SP wrote the manuscript with input from all authors.

## Supplementary Material

Supplemental data

## Version 1 03/01/2022

In-Press Preview

## Version 2 04/08/2022

Electronic publication
