# Supplementary material for: In-depth analysis of SARS-CoV-2–specific T cells reveals diverse differentiation hierarchies in vaccinated individuals
Source: JCI Insight. 2022 Apr 8;7(7):e156559. doi: 10.1172/jci.insight.156559 (PMC9057595; doi:10.1172/jci.insight.156559)
Supplement: Supplemental data [file jciinsight-7-156559-s249.pdf]

Supplemental table 1. Donor characteristic

Status	Gender	Age	Vaccine brand	Days after last dose	Adverse effects
Donor #1	M	56	Pfizer	90.00	Muscle aches, fatigue
Donor #2	M	32	Johnson&Johnson	67.00	Muscle aches, fatigue
Donor #3	F	25	Pfizer	103.00	Muscle aches, fatigue
Donor #4	M	43	Moderna	95.00	Muscle aches, fatigue
Donor #5	F	41	Moderna	110.00	Muscle aches, fatigue
Donor #6	F	43	Pfizer	110.00	Muscle aches, fatigue
Donor #7	M	24	Moderna	108.00	Muscle aches, fatigue
Donor #8	F	38	Moderna	108.00	Mild fever, muscle aches, fatigue
Donor #9	M	40	Moderna	108.00	Muscle aches, fatigue
Donor #10	F	30	Pfizer	105.00	Muscle aches, fatigue
Donor #11	F	50	Moderna	108.00	Fever, muscle aches, fatigue
Donor #12	M	25	Moderna	110.00	Fever, muscle aches, fatigue
Donor #13	F	36	Moderna	110.00	fever, muscle aches, fatigue, chills in the pain
Donor #14	M	30	Pfizer	51.00	Muscle aches, fatigue
Donor #15	M	25	Moderna	110.00	Muscle aches, fatigue
Donor #16	M	26	Moderna	106.00	Muscle aches, fatigue, fever
Donor #17	M	26	Pfizer	78.00	Muscle aches, fatigue
Donor #18	M	25	Pfizer	13.00	Muscle aches, fatigue
Donor #19	M	45	Moderna	110.00	muscle aches, fatigue
Donor #20	F	46	Pfizer	105.00	Fever, Headache, muscle aches, fatigue
Donor #21	M	42	Pfizer	105.00	Fever, Headache, muscle aches, fatigue

Supplemental table 2. Sample collection date from healthy donors

Samples	Collection Date
PBMC1	5-28-2019
PBMC2	6-28-2019
PBMC3	7-25/2019
PBMC4	7-25/2019
PBMC5	7-25/2019
PBMC6	6/18/18
PBMC7	6/18/18
PBMC8	6/18/18
PBMC9	7/2/18
PBMC10	7/2/18
PBMC11	7/2/18
PBMC12	2-20-19
PBMC13	9/3/19
PBMC14	10/5/19
PBMC15	8/1/19
PBMC16	12/1/18
PBMC17	12/1/18

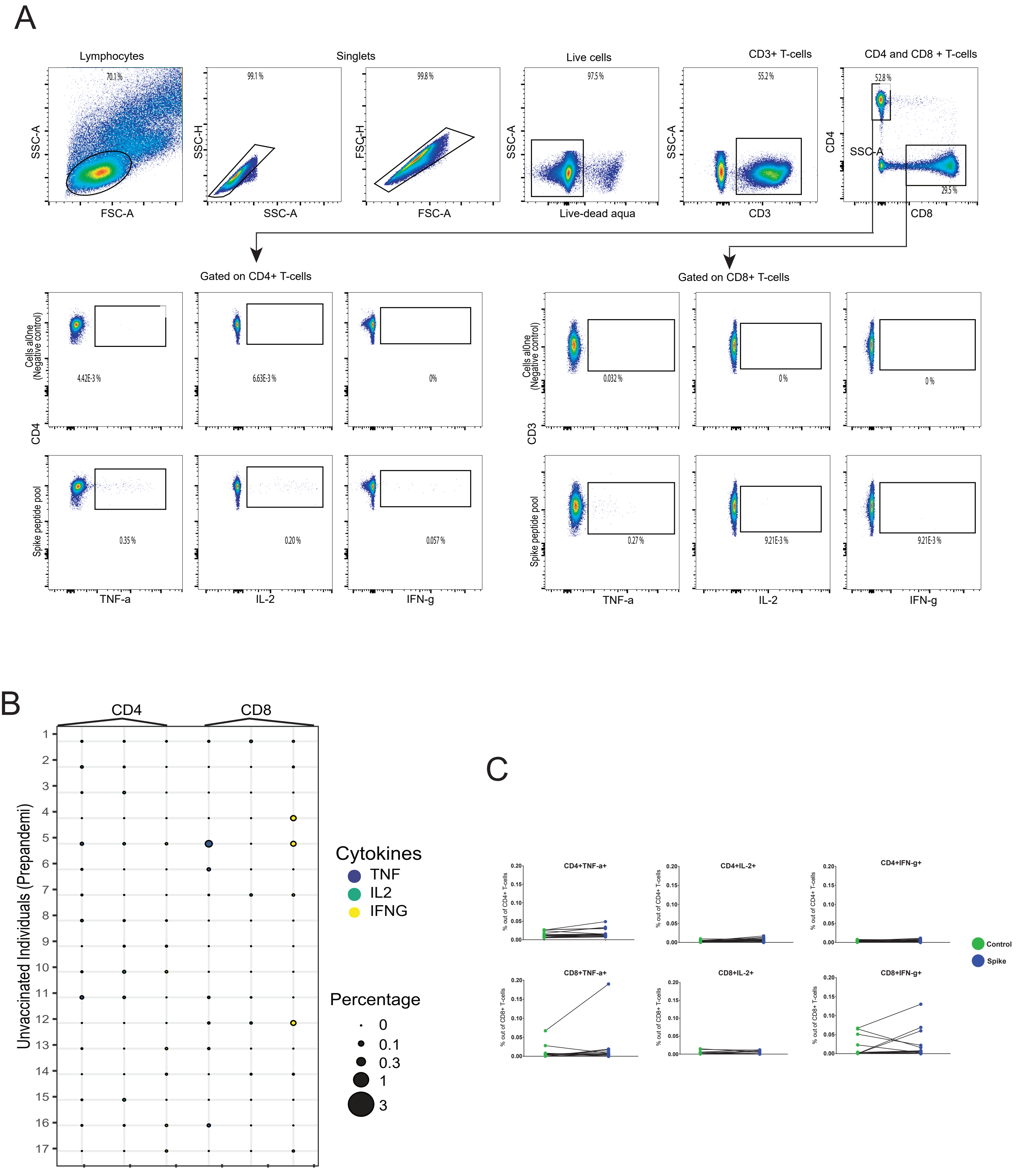

Supplemental figure 1. (A)Gating strategy for quantification of SARS-CoV-2 specific T-cells. Representative FACS plots show gating strategy utilized to calculate the frequencies of cytokine secreting CD4+ and CD8+ T-cells (donor #). (B) The bubble plot shows the percentage of TNF-a, IL-2 and IFN-g secreting CD4+ and CD8+ T-cells isolated from 17 healthy exposed individuals after coculturing with S-protein peptide pool. The colours indicate different cytokines detected in response to stimulation and the node size represents percentages of cytokine secreting CD4+ and CD8+ T-cells. (C) Cytokine secreting CD4+ (upper panel) and CD8+ (lower panel) T-cell frequencies in healthy unexposed individuals. Cytokine secretion levels after culturing in media alone and S-protein peptide pools are shown.

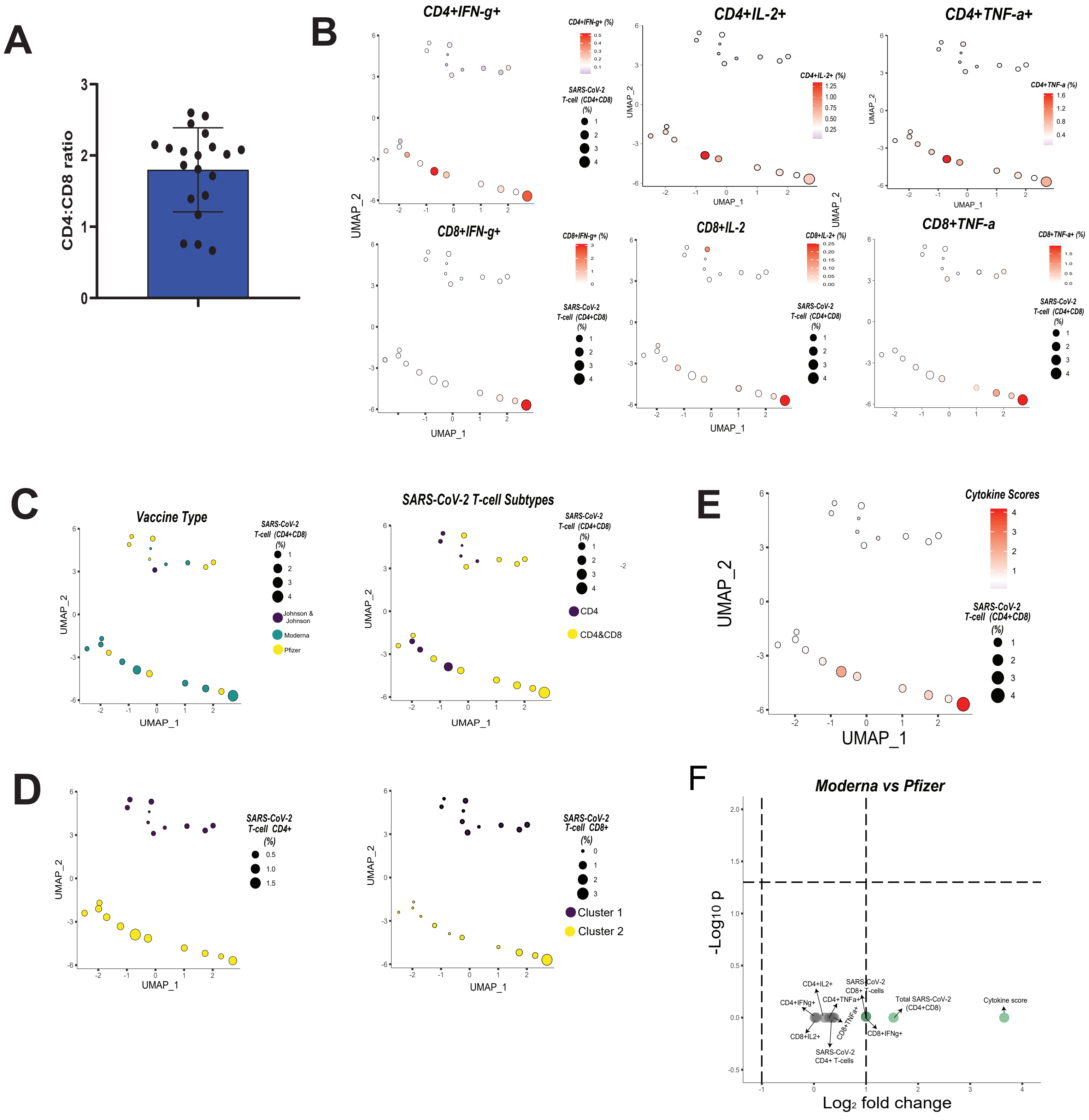

Supplemental figure 2. (A) Bar plot shows the CD4:CD8 ratio in twenty-one vaccinated individuals. (B) The UMAP plots were generated using the frequencies of cytokine secreting CD4+ and CD8+ T-cells following stimulation WT S-protein peptide pool. The UMAP plots are colored for CD4+IFN-g+ (%) (upper left), CD8+TNF-a+ (%) (upper right), CD8+IFN-g+ (%) (lower left) and CD8+IL-2+ (%) (lower right). The color indicates SARS-CoV-2 T-cells producing the indicated cytokines. The bubble size indicates the SARS-CoV-2 T-cell frequencies (CD4+ and CD8+ combined) (related to Figure 2D). (C) The UMAP plots are colored vaccine types (left) and vaccine-induced SARS-CoV-2 response patterns (right), yellow color was used to denote individuals having both SARS-CoV-2 CD4+ and CD8+ T-cells and purple color indicates individuals with SARS-CoV-2 CD4+ T-cell response only after vaccination. The bubble size indicates the percentage of SARS-CoV-2 T-cells. (D) The UMAP plots show the frequencies of SARS-CoV-2 CD4+ (left) and CD8+ (right) T-cells across cluster 1 (low responders) and cluster 2 (high responders). The size indicates the percentage of SARS-CoV-2 T-cells. (E) The UMAP plot shows the cytokine score across twenty-one vaccinated individuals (top). The cytokine scores were calculated by summing the frequencies of IL2, TNF-a and IFN-g producing CD4+ and CD8+ T-cells. The volcano plot (down) shows the comparative analysis of CD4+ and CD8+ T-cell originated cytokine expression analysis in individuals vaccinated with Moderna vs Pfizer vaccine. The dashed lines indicate the statistical cut-off for p value ≤0.05 (y-axis) and a cut-off value of 1 (dashed line) was arbitrarily chosen to show relative fold changes (right). Gray color indicates fold change <1 and green color indicates fold change >1.

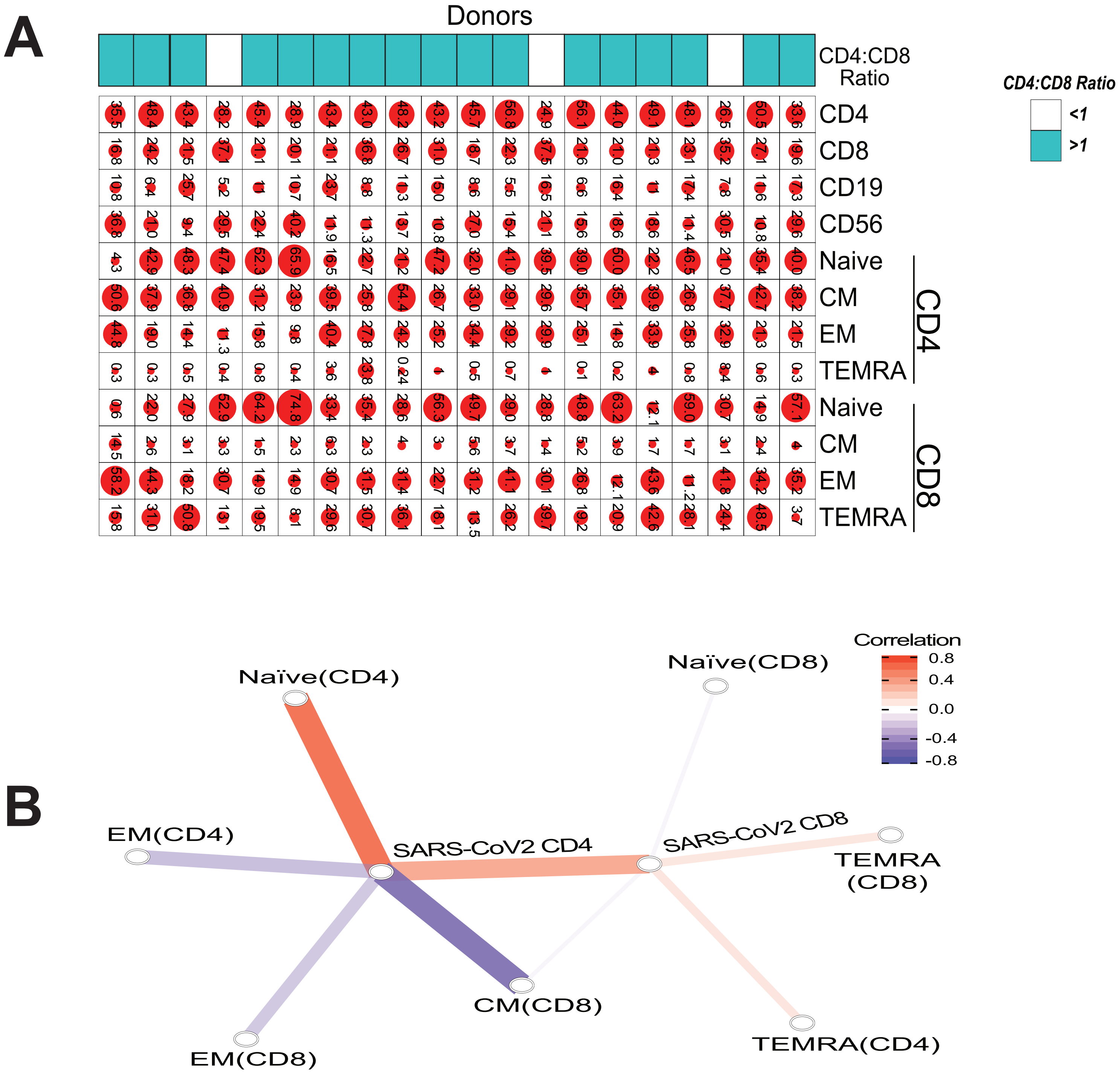

Supplemetnal figure 3. (A) Dot plot shows the frequencies of multiple immune features. The frequencies were calculated using FlowJo with manual gating. The circle size indicates the percentage of each subset across all the individuals and the actual frequencies used to infer circle size are shown for each condition. The color bar is used to show CD4:CD8 ration. White color indicates CD4:CD8 ration <1 and cyan color indicates CD4:CD8 ratio > 1. (B) The significant associations detected in Figure 2F are plotted using correlation network plot. Red and blue colors indicate positive and negative associations, respectively. The line thickness indicates the degree of association. The thicker lines correspond to a higher association.

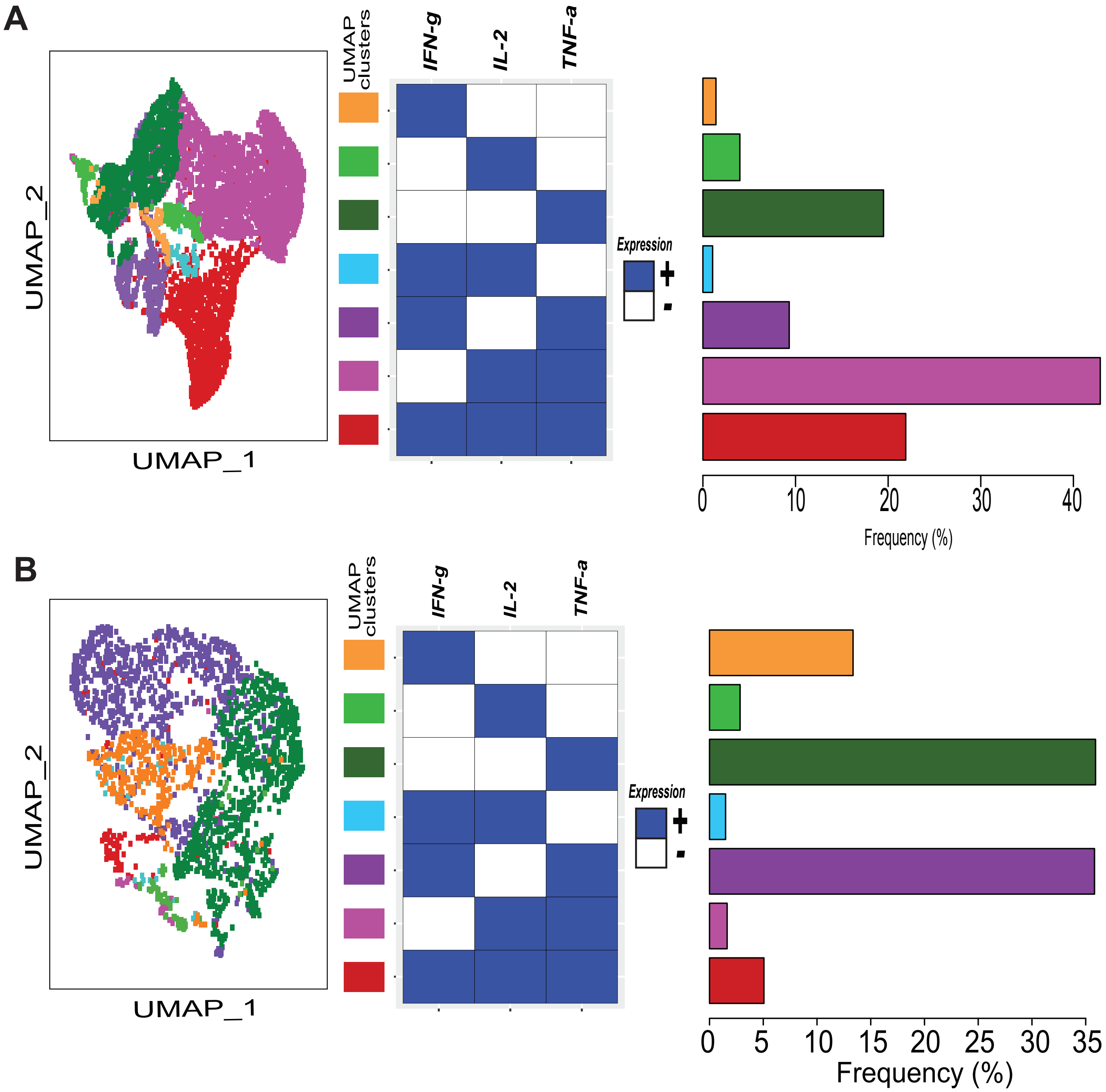

Supplemental figure 4. (A) UMAP plot shows cytokine producing SARS-CoV-2 specific CD4+ T cells, color bar indicates the cytokine combination. The frequency of each combination was shown in the bar plot (right). (B). UMAP plot shows cytokine producing SARS-CoV-2 specific CD8+ T cells, color bar indicates the cytokine combination. The frequency of each combination was shown in the bar plot (right).

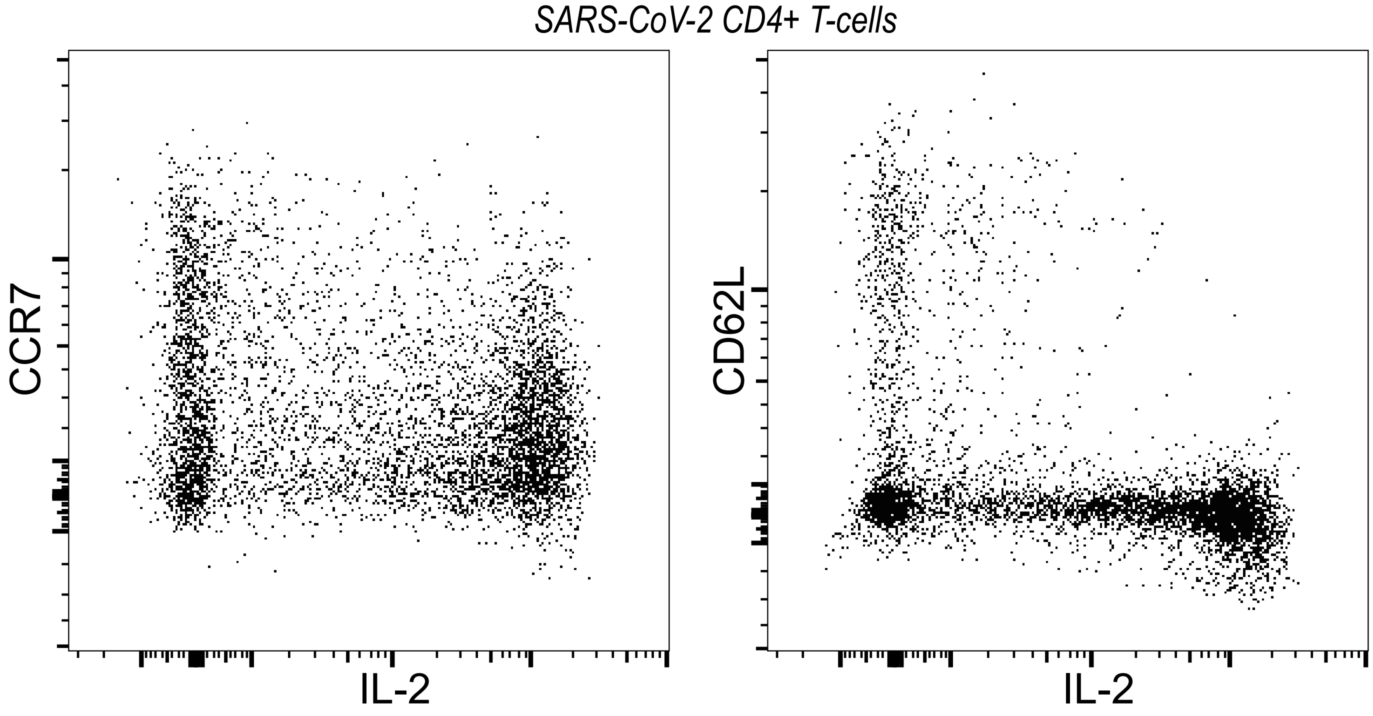

Supplemental figure 5. The correlation between the expression of IL2 and CCR7(left), IL2 and CD62L(right) in SARA-CoV-2 specific CD4+ T cells .

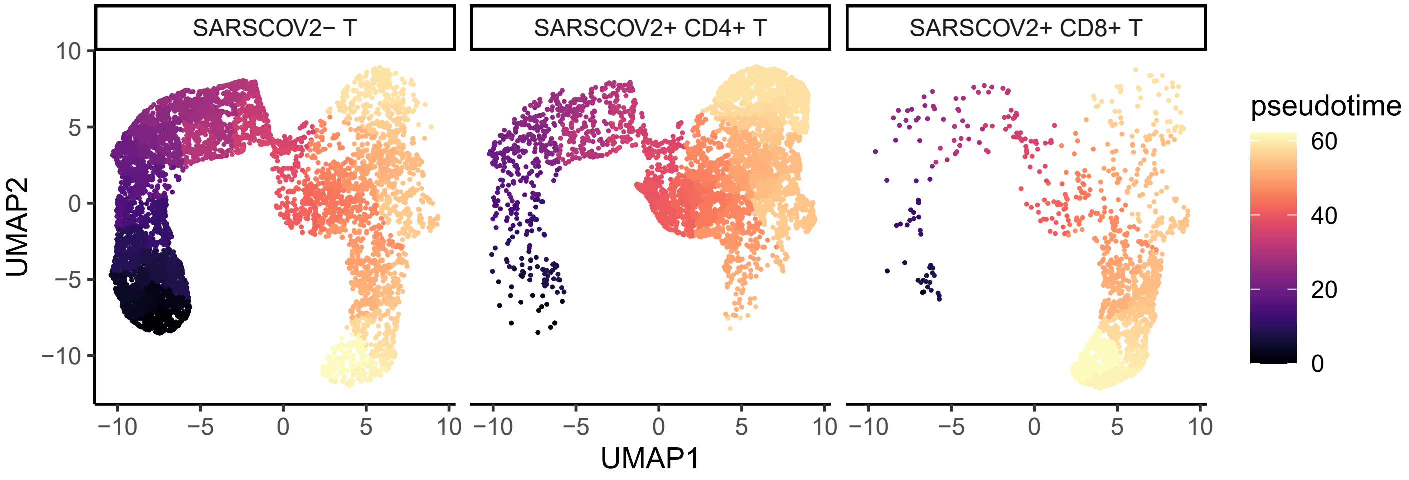

Supplemental figure 6.SARS-CoV-2 specific CD4+ (middle) and CD8+ (right) T cells and equal number of SARS-CoV-2 (-) T-cells (left) were pooled subjected to UMAP dimension reduction and differentiation state of each single-cell was inferred through pseudo-time analysis using monocle 3. Pseudo-time values are shown for each single cell.

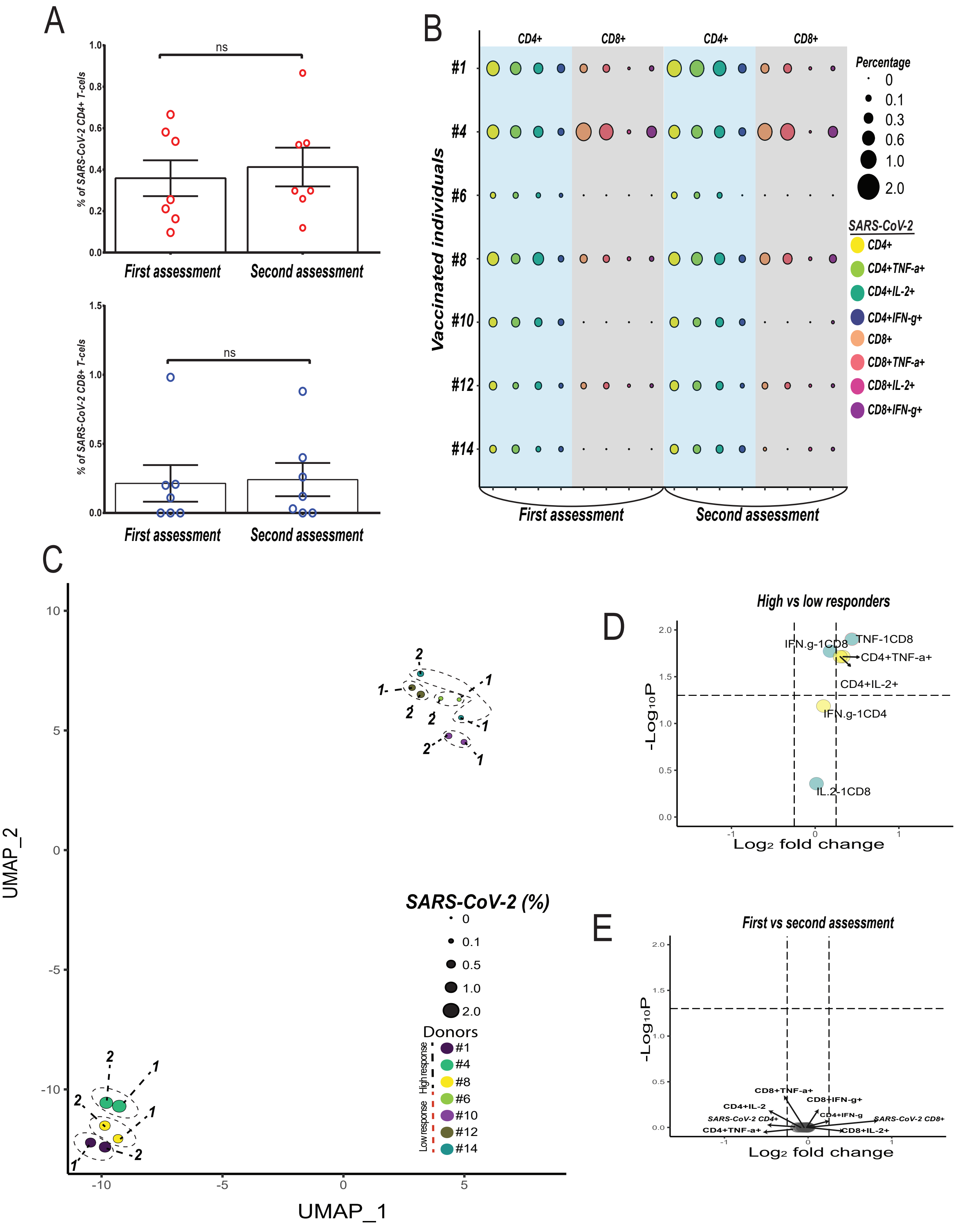

Supplemental figure 7. SARS-CoV-2 vaccines elicit a stable SARS-CoV-2 specific immune response. (A) Bar plots show the fractions of SARS-CoV-2 specific CD4+ (top) and CD8+ (bottom) assessed at two different timepoints after vaccination in seven individuals. (B) The bubble plot shows the percentage of TNF-a, IL-2 and IFN-g secreting CD4+ and CD8+ T-cells and the frequencies of SARS-CoV-2 specific CD4+ and CD8+ T-cells in seven vaccinated individuals at two different timepoints. The colors indicate cytokines and the size represents cytokine percentage and SARS-CoV-2 specific CD4 or CD8+ T-cells frequencies. (C) UMAP plot shows differential response to vaccination in seven individuals. The nodes representing the early timepoints are labeled as “1” and late timepoints as “2”. Each color represents a single donor and the node size indicates the fractions of SARS-CoV-2 specific T-cells at indicated timepoints. (D) Volcano plot shows the differentially expression of cytokines between high and low responders. (E) Volcano plot shows the differentially expression of cytokines between first and second assessment (n=7).
